# Sea snakes (Elapidae, Hydrophiinae) in their westernmost extent: an updated and illustrated checklist and key to the species in the Persian Gulf and Gulf of Oman

**DOI:** 10.3897/zookeys.622.9939

**Published:** 2016-10-06

**Authors:** Mohsen Rezaie-Atagholipour, Parviz Ghezellou, Majid Askari Hesni, Seyyed Mohammad Hashem Dakhteh, Hooman Ahmadian, Nicolas Vidal

**Affiliations:** 1 Environmental Management Office, Qeshm Free Area Organization, Qeshm Island, P. O. Box 7951614465, Hormozgan Province, Iran; 2 Department of Phytochemistry, Medicinal Plants and Drugs Research Institute, Shahid Beheshti University, Tehran, Iran; 3 Department of Biology, Faculty of Sciences, Shahid Bahonar University of Kerman, Kerman Province, Iran; 4 Green Reef Group, Qeshm Island, Hormozgan Province, Iran; 5 ISYEB, UMR7205 MNHN-CNRS-UPMC-EPHE, Muséum national d’Histoire naturelle, Département Systématique et Evolution, CP30 25 rue Cuvier 75005 Paris, France

**Keywords:** Indian Ocean, new record, reptiles, Serpentes, venomous, viviparous

## Abstract

The Persian Gulf is known as the westernmost distribution limit for sea snakes, except for *Hydrophis platurus* (Linnaeus, 1766) that reaches southeastern Africa. Previous identification guides for sea snakes of the Persian Gulf and its adjacent waters in the Gulf of Oman were based on old data and confined mostly to written descriptions. Therefore, a series of field surveys were carried out in 2013 and 2014 through Iranian coastal waters of both gulfs to provide a comprehensive sampling of sea snakes in the area. This paper presents an illustrated and updated checklist and identification tool for sea snakes in the Persian Gulf and Gulf of Oman, which are based on new material and a review of the literature. This checklist includes ten species of marine hydrophiines, of which one, *Microcephalophis cantoris* (Günther, 1864), is a new record for the area. All specimens examined herein are deposited and available at the Zoological Museum of Shahid Bahonar University of Kerman, Kerman province, Iran.

## Introduction

All true sea snakes of the subfamily Hydrophiinae share a common ancestor dating back to approximately six million years ago, although the majority of the extant lineages have diversified in the last three and half million years (Sanders et al. 2013). Now, more than 60 morphologically and ecologically diverse species of highly venomous marine hydrophiines live throughout tropical and subtropical coastal waters of the Indo-West Pacific region (Rasmussen et al. 2011b), with an exception, the Yellow-bellied Sea Snake, *Hydrophis platurus* (Linnaeus, 1766), that lives in both Indian and Pacific Oceans (Heatwole 1999). These sea snakes colonize various coastal habitats throughout their geographic range and play an important role in the food web of these coastal biomes by consuming various prey (Voris 1972).

The Persian Gulf is a semi-enclosed shallow marine environment (mean depth ca. 35 meters) lying in a subtropical and hyper-arid region in the northwestern Indian Ocean. This water body is considered a young sea (~15,000 years) with impoverished species biodiversity. Biota living in the Gulf must adapt to high temperatures and a hypersaline environment (Price 2002; Sheppard 1993; Sheppard et al. 2010). Sea surface temperature in the Gulf varies from 18 to 34°C throughout the year and salinity is more than 39 ppt in most areas (Sheppard et al. 2010).

Populations of sea snakes in the Persian Gulf are peculiar for two main reasons. First, because the Gulf is known as the westernmost extent of sea snakes (with the exception of *Hydrophis platurus*, which is also found in the east coast of Africa) (Heatwole 1999). Second, because the Persian Gulf can be considered as an excellent natural laboratory to study the adaptive responses of the rapidly evolving sea snakes to high salinities and fluctuating temperatures.

Nonetheless, our knowledge about the sea snake diversity in the Persian Gulf and its adjacent waters is based on older studies (e.g. Smith 1926; Volsøe 1939), documenting the occurrence of nine species of the subfamily Hydrophiinae in the area. Recently, taxonomy of the true sea snakes has been revised based on comprehensive molecular phylogenetic analyses (Sanders et al. 2013). After Sanders et al. (2013), sea snake species from the Persian Gulf and Gulf of Oman previously allocated to the genera *Enhydrina* Gray, 1849, *Lapemis* Gray, 1835 and *Pelamis* Daudin, 1803 are now all assigned to the single genus *Hydrophis* Latreille *in* Sonnini & Latreille, 1801; and the Small-headed Sea Snake previously known as *Hydrophis gracilis* (Shaw, 1802) is now assigned to the genus *Microcephalophis* Lesson, 1834. Furthermore, our new material examination confirmed the occurrence of a second species of Small-headed Sea Snake, namely *Microcephalophis cantoris* (Günther, 1864), in the Gulf of Oman. Therefore, the checklists and identification keys for the sea snakes in the gulfs (e.g. Egan 2007; Gasperetti 1988; Leviton et al. 1992) must be revised and updated by examining new material and using updated taxonomic classification (e.g. Sanders et al. 2013). This paper aims at presenting an illustrated and up to date checklist for sea snakes in the Persian Gulf and Gulf of Oman, as well as easy to use identification keys to the genera and species recorded in both gulfs.

## Materials and methods

**Figure 1.**
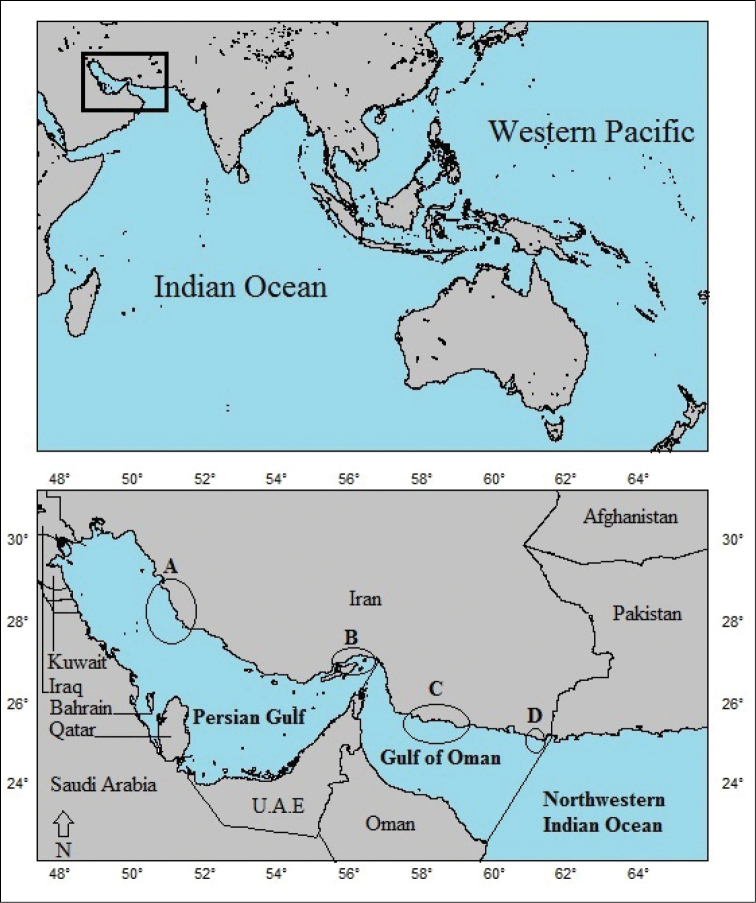
The upper map shows location of the Persian Gulf and Gulf of Oman in the Indo-West Pacific; the lower map shows study sites in the gulfs, including: **A** Bushehr Province **B** Qeshm Islands (Qeshm, Larak, Hormouz and Hengam Islands) **C** Jask and Ras-Meydani **D** Beris and Pasa-Bandar.

The sea snakes examined herein were collected from the Iranian coastal waters of the Persian Gulf and Gulf of Oman (Figure 1). In this study, boundaries of both gulfs were assumed following International Hydrographic Organization (IHO). According to the descriptions of the organization, the Persian Gulf is separated from the Gulf of Oman by an imaginary line from Minab (27°00'N, 57°00'E) on the Iranian coast to Ras Qabr al-Hindi (26°20'N, 56°30'E) on the northeast tip of the Musandam Peninsula. Furthermore, the eastern limit of the Gulf of Oman is an imaginary line running from Ras Jiwani (25°01'N, 61°44'E) on the border of Pakistan and Iran to Ras al-Hadd (22°32'N, 59°47'E) in Oman (Figure 1).

Sea snakes were collected from by-catch of fishing trawlers in coastal waters of Bushehr Province in the western Persian Gulf, Jask and Ras-Meydani (Hormozgan Province) in the western Gulf of Oman and Beris and Pasa-Bandar (Sistan-O-Baluchestan Province) in the eastern Gulf of Oman in 2013 (Figure 1). Sea snakes were also collected from mangrove swamps of Jask (Hormozgan Province) in the western Gulf of Oman in 2013, and coastal waters of Larak Island (Hormozgan Province) in the eastern Persian Gulf in 2014 (Figure 1) using boat surveys.

A total of 14 characters was examined: each specimen was measured for total length (TL), snout-vent length (SVL), head length (HL), head width (HW), gap length (GL), snout to nostril length (SNL), nostril to eye length (NEL), neck diameter (ND) and greatest body diameter (GBD). Furthermore, number of supralabials (NSL), number of scale rows on the neck (NSR), number of scale rows on the body (BSR), number of ventrals (NV), and number of bands (NB) were also recorded for each specimen. SVL, HW, GL, SNL and NEL were measured following Ukuwela et al. (2012). HL was measured following Thorpe (1975). NSR and BSR were counted following Rasmussen (2001). NV was counted following Dowling (1951). After detailed morphological examination, a piece of trunk muscle was removed and preserved in ethanol 99% for future DNA analyses. All specimens were then preserved and fixed using the approved protocols (Leviton et al. 1992) and deposited in the Zoological Museum of Shahid Bahonar University of Kerman, Kerman Province, Iran (institutional code: ZMSBUK.HD).

Some external diagnostic characters (e.g. coloration patterns, number, size and shape of head shields, and body and ventral scales) were used to make updated and easy to use identification keys, which allow users to identify sea snakes in the field without the need of a binocular.

Morphological characters are presented using abbreviations (see above). Data of all measurements are in mm. Due to great interspecific and intraspecific variations in external characters of sea snakes (Rasmussen et al. 2011), diagnostic characters provided here are mostly applicable to the specimens from this area. Morphological data derived from previous literature dealing with sea snakes in the area are mentioned in brackets. Synonymies previously used for each species in the region are listed under each species. English common names presented herein are those suggested by the IUCN Red List of threatened species (IUCN 2016). Persian (Farsi) common names presented herein for the species in the genus *Hydrophis* are those suggested by Firouz (2005). For the two species in the genus *Microcephalophis*, English common names were directly translated into the Persian and presented herein as Persian common names.

## Results

### Key to the genera of the subfamily Hydrophiinae in the Persian Gulf and Gulf of Oman

**Table:** 

1	Head extremely small; neck markedly slender; usually 5–6 supralabials; usually less than 25 scale rows on neck (Figures 23–26)	***Microcephalophis***
–	Head and neck not as in 1; usually more than 6 supralabials; usually more than 25 scale rows on neck (Figures 5–22)	***Hydrophis***

### Key to the species of the genus *Hydrophis* in the Persian Gulf and Gulf of Oman

**Table:** 

1	Tip of rostral decurved and pointed (beaked-shaped; Figures 2a and 3a); mental shield narrow and elongate (dagger-shaped), hidden in groove between chin shields (Figures 3a and 6c) (Figures 5–7)	***Hydrophis schistosus***
–	Tip of rostral curved and dentate (Figures 2b–d, 3b–h); mental shield short and triangulate (Figure 3b–h)	**2**
2	Tip of rostral markedly tridentate (Figures 2b, 2c, 3b, 3c); ventrals on mid-body larger anteriorly than posteriorly, markedly distinguishable from adjacent scales (Figure 4a–b)	**3**
–	Tip of rostral markedly or slightly unidentate (Figures 2d, 3d-h); ventrals on mid-body almost of same size, slightly distinguishable or indistinguishable from adjacent scales (Figure 4c–f)	**4**
3	Ventrals on anterior part of body markedly large, wide and rectangular in shape (Figure 4a), half width of body (Figures 8–9)	***Hydrophis viperinus***
–	Ventrals on anterior part of body medium size, more or less hexagonal in shape (Figure 4b), less than half width of body (Figures 10–11)	***Hydrophis curtus***
4	Markedly sharp contrast in colors of dorsal and ventral portions of head and body, dark brown or black dorsally, yellow ventrally (Figure 13d); ventrals on mid-body more or less indistinguishable from adjacent scales (Figure 4f) (Figures 12–13)	***Hydrophis platurus***
–	Not colored as in 4, usually banded body (Figures 15d, 17d–e, 19d, 20, 22); ventrals on mid-body slightly distinguishable from adjacent scales (Figure 4c–d)	**5**
5	Body bands narrower than light interspaces (Figure 15d) (Figures 14–15)	***Hydrophis spiralis***
–	Body bands as wide as or wider than light interspaces (Figures 17d–e, 19d, 20, 22)	**6**
6	Head slightly small; body elongate; body bands broader dorsally tapering to points in lateral sides (Figures 19d, 20, 22); usually less than 32 scale rows on neck	**7**
–	Head of medium size; body slightly stout; rhomboidal or rectangular dark body bands clearly distinct with light narrow interspaces (Figure 17); usually more than 34 scale rows on neck (Figures 16–17)	***Hydrophis ornatus***
7	Scales on thickest part of body juxtaposed or feebly imbricate, more or less hexagonal or quadrangular in shape; total length rarely exceeding one meter in adults (Figures 21–22)	***Hydrophis lapemoides***
–	Scales on thickest part of body more or less imbricate with bluntly pointed tips; total length more than one meter in adults (Figures 18–20)	***Hydrophis cyanocinctus***

**Figure 2.**
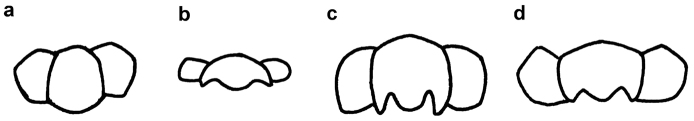
Various shapes of rostrals of sea snakes of the genus *Hydrophis* in the Persian Gulf and Gulf of Oman: **a** tip of rostral decurved and pointed (beaked-shaped) **b** and **c** tip of rostral tridentate **d** tip of rostral unidentate.

**Figure 3.**
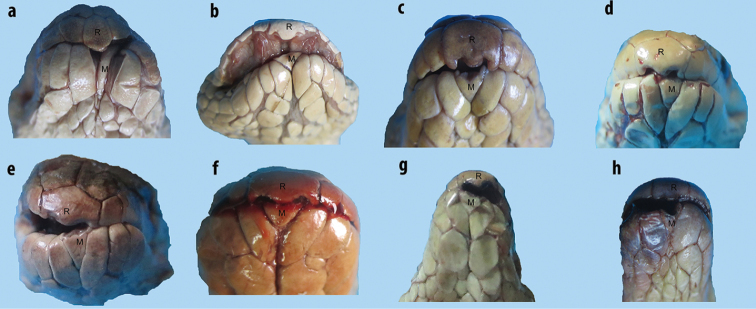
Rostrals and mentals of sea snakes of the genus *Hydrophis* in the Persian Gulf and Gulf of Oman: **a** tip of rostral decurved, pointed and beaked shaped, mental narrow, elongate and dagger-shaped (*Hydrophis schistosus*) **b** and **c** tip of rostral markedly tridentate, mental short and triangulate (*Hydrophis viperinus* and *Hydrophis curtus*, respectively) **d** tip of rostral markedly unidentate, mental short and triangulate (*Hydrophis ornatus*) **e–h** tip of rostral slightly unidentate, mental short and triangulate (*Hydrophis ornatus*, *Hydrophis spiralis*, *Hydrophis cyanocinctus* and *Hydrophis platurus*, respectively).

**Figure 4.**
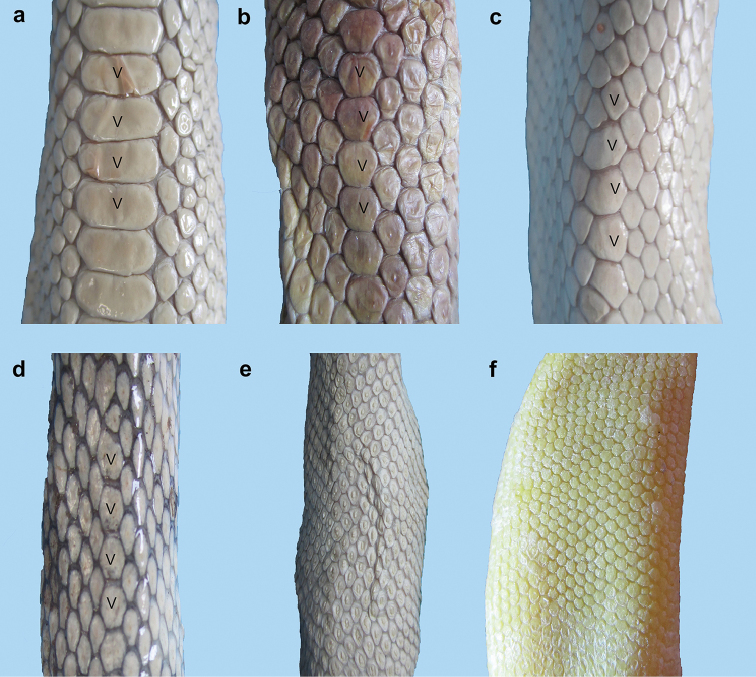
Various shapes of ventrals on the mid-body of sea snakes of the genus *Hydrophis* in the Persian Gulf and Gulf of Oman: **a** wide and enlarged, more or less rectangular in shape (*Hydrophis viperinus*) **b** medium size, distinguishable from the adjacent scales, more or less hexagonal in shape (*Hydrophis curtus*) **c** and **d** small and slightly distinguishable from adjacent scales (*Hydrophis ornatus* and *Hydrophis lapemoides*, respectively) **e** and **f** small and more or less indistinguishable from adjacent scales (*Hydrophis schistosus* and *Hydrophis platurus*, respectively).

**Figure 5.**
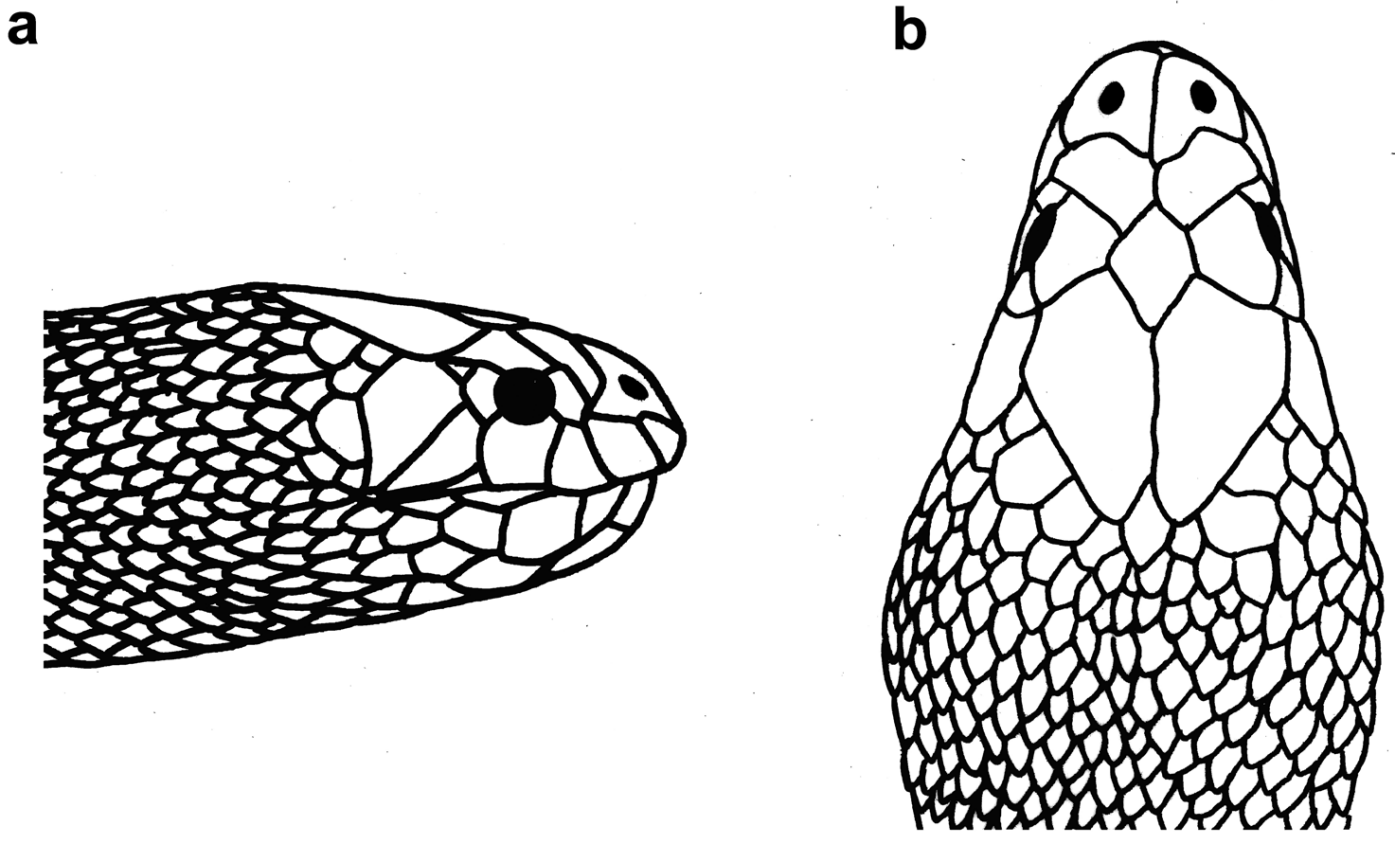
Head of *Hydrophis schistosus*: **a** lateral view **b** dorsal view.

**Figure 6.**
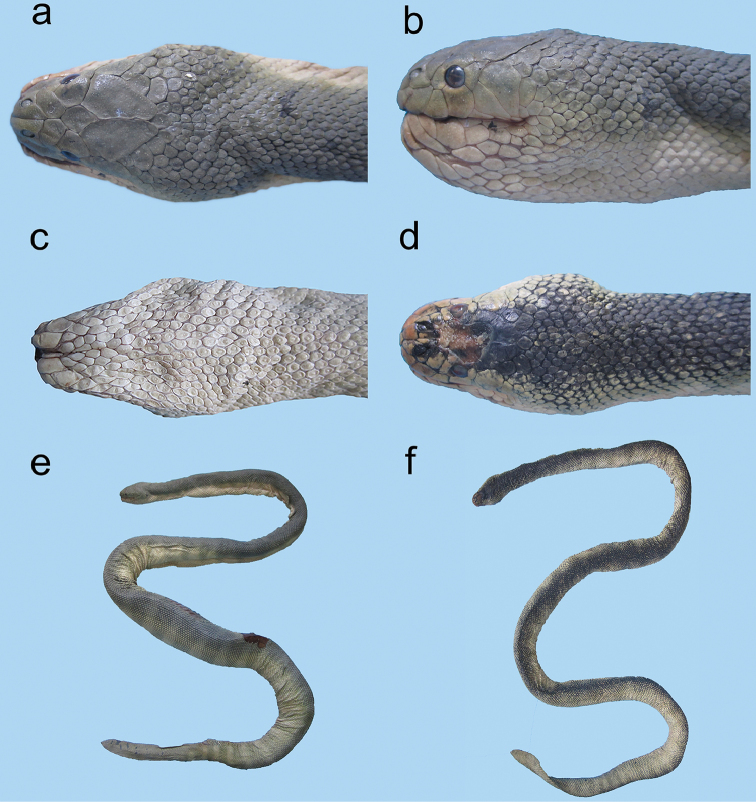
*Hydrophis schistosus*: **a** dorsal view **b** lateral view, and **c** ventral view of head **d** dorsal view of head of a black specimen **e** typical body color **f** black dorsally.

**Figure 7.**
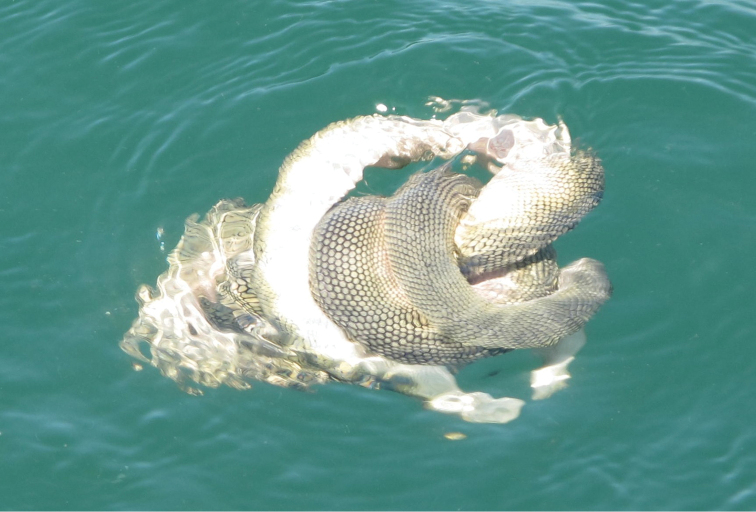
Mating behavior of *Hydrophis schistosus*: two individuals intertwined each other and floating on the surface in the coastal waters of Jask (western Gulf of Oman), December 2013.

**Figure 8.**
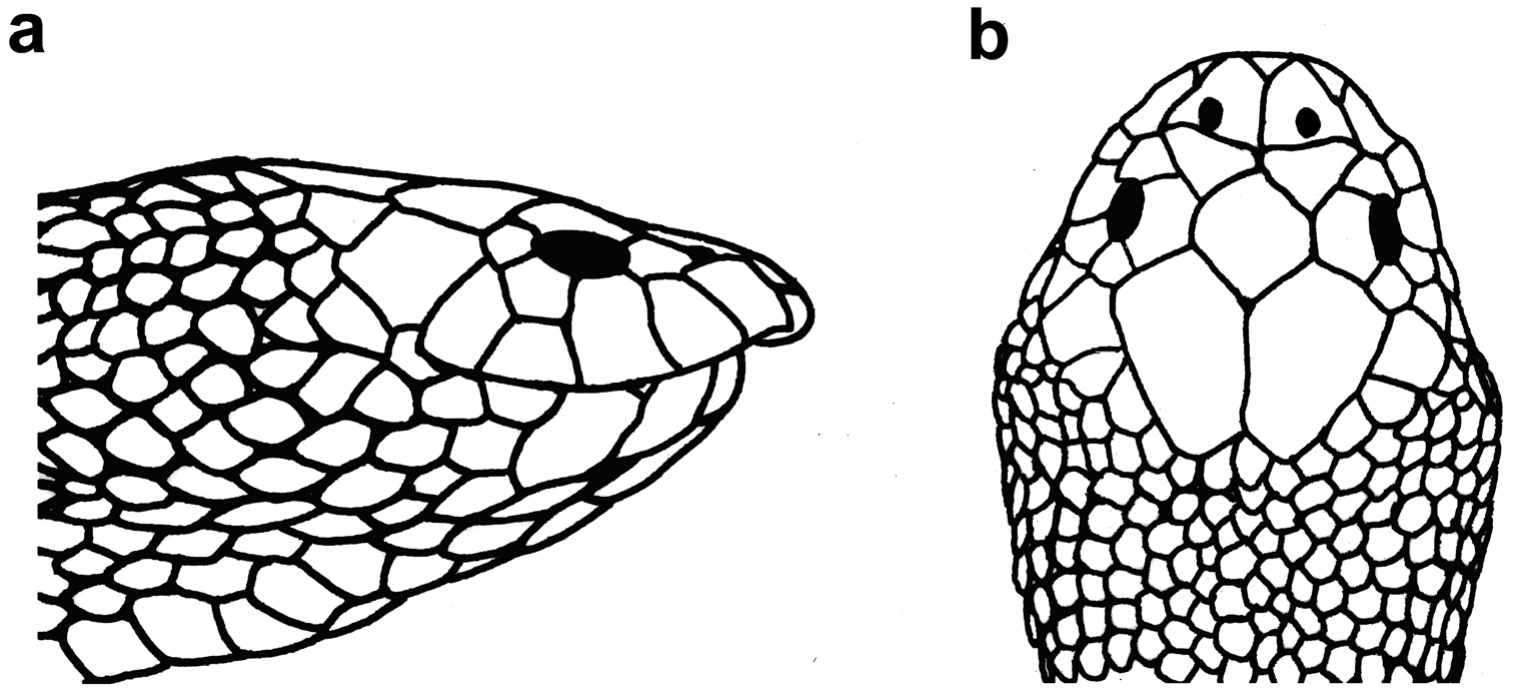
Head of *Hydrophis viperinus*: **a** lateral view **b** dorsal view.

**Figure 9.**
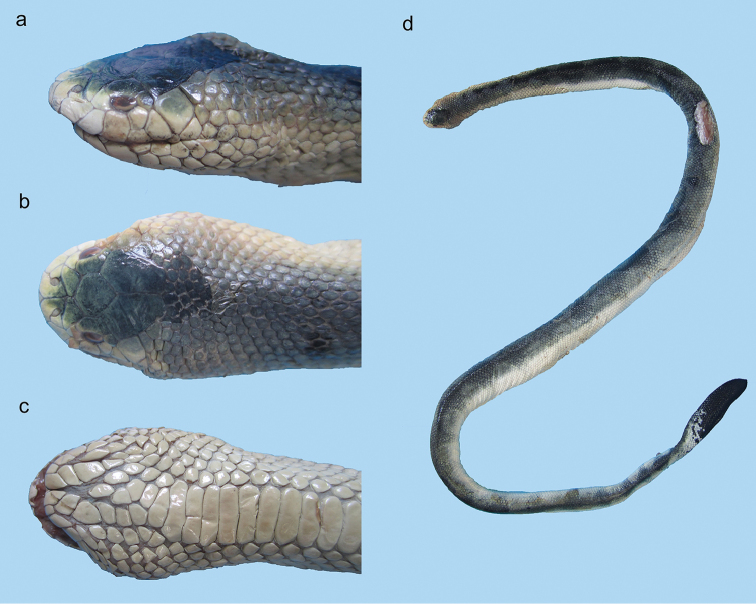
*Hydrophis viperinus*: **a** lateral view **b** dorsal view, and **c** ventral view of head **d** body.

**Figure 10.**
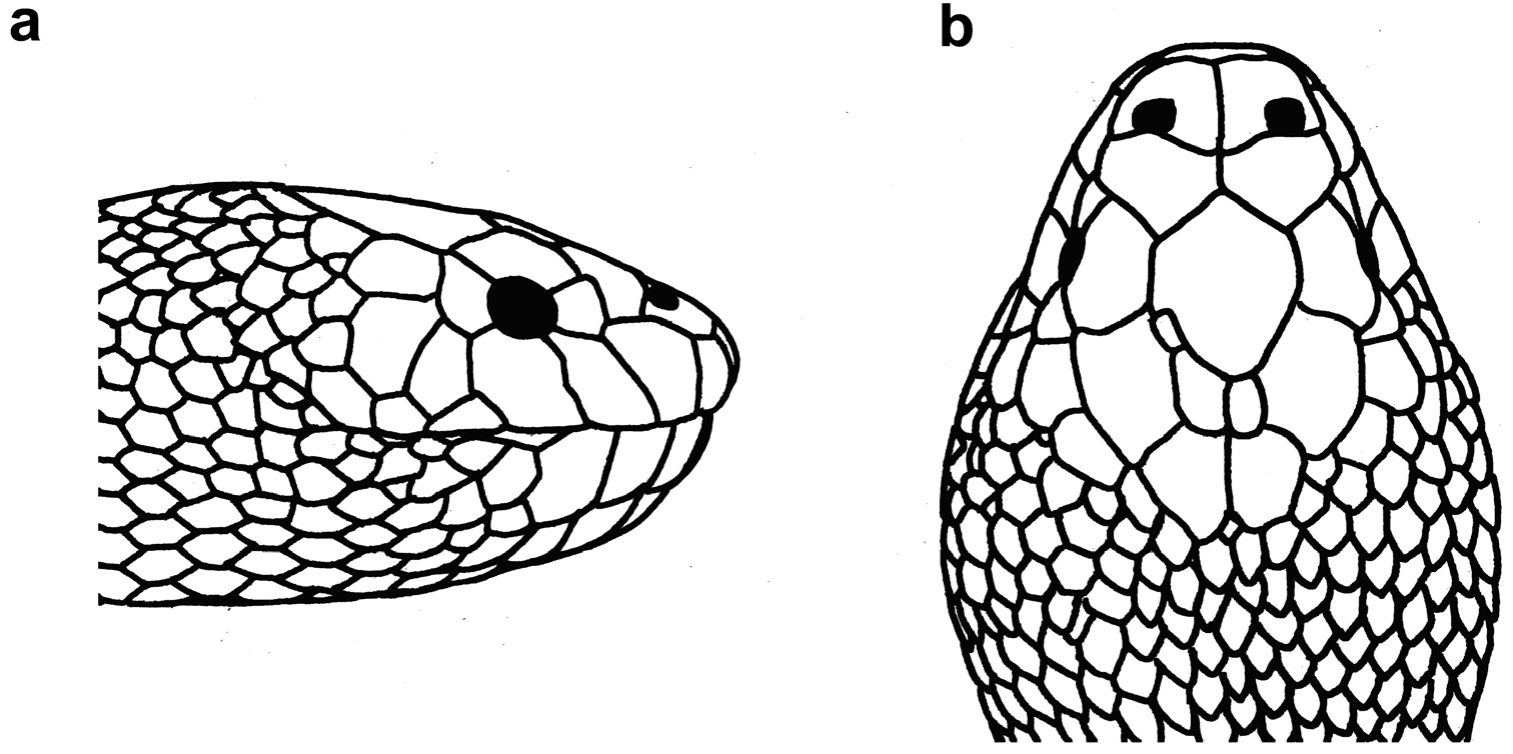
Head of *Hydrophis curtus*: **a** lateral view **b** dorsal view.

**Figure 11.**
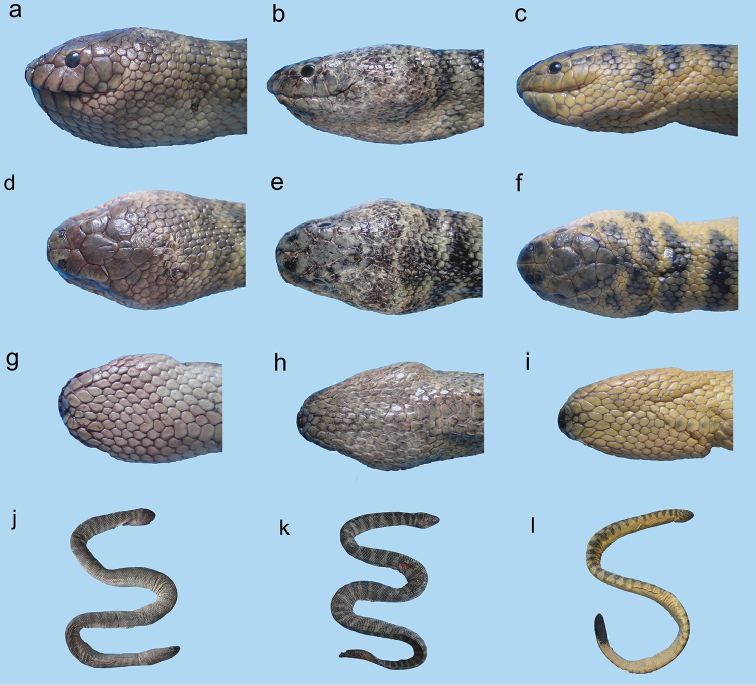
*Hydrophis curtus*: **a, b, c** lateral view **d, e, f** dorsal view **g, h, i** ventral view of head; and **j, k, l** body of a typical gray specimen, a rare black specimen and a rare yellow specimen, respectively.

**Figure 12.**
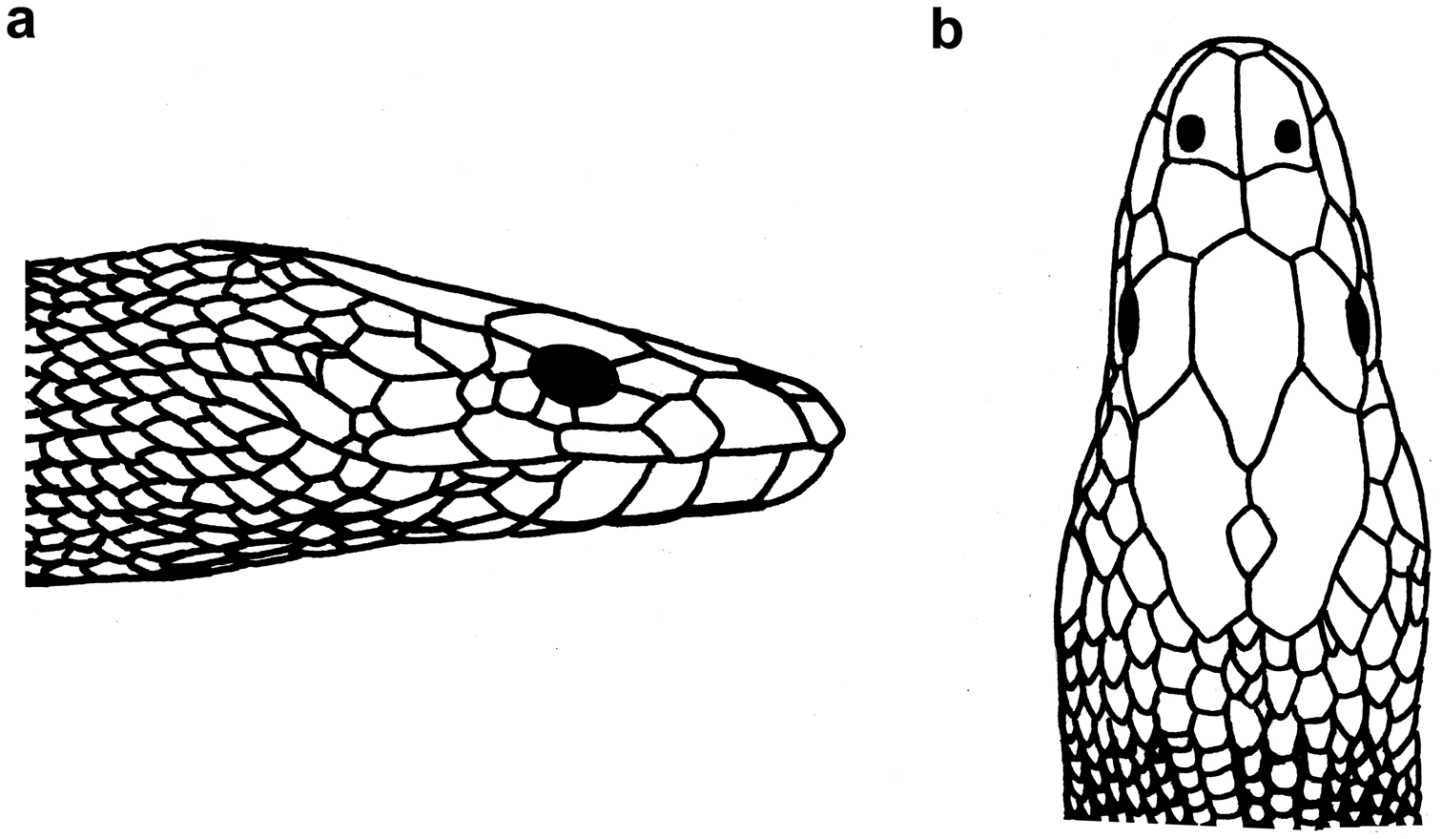
Head of *Hydrophis platurus*: **a** lateral view **b** dorsal view.

**Figure 13.**
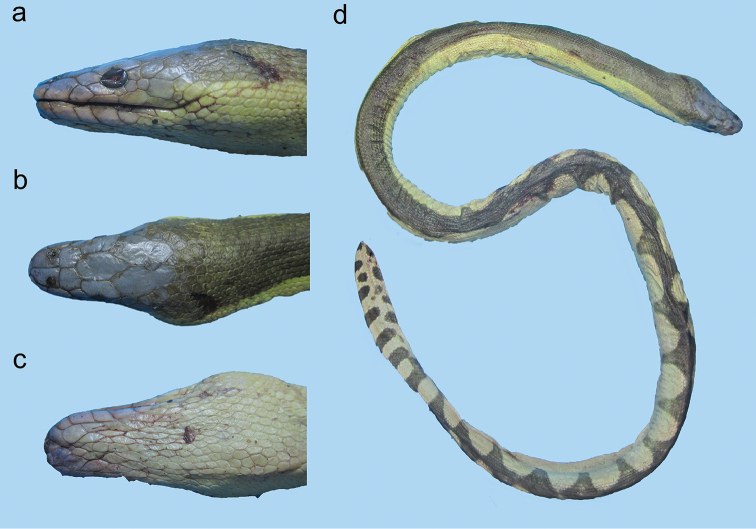
*Hydrophis platurus*: **a** lateral view **b** dorsal view, and **c** ventral view of head **d** body.

**Figure 14.**
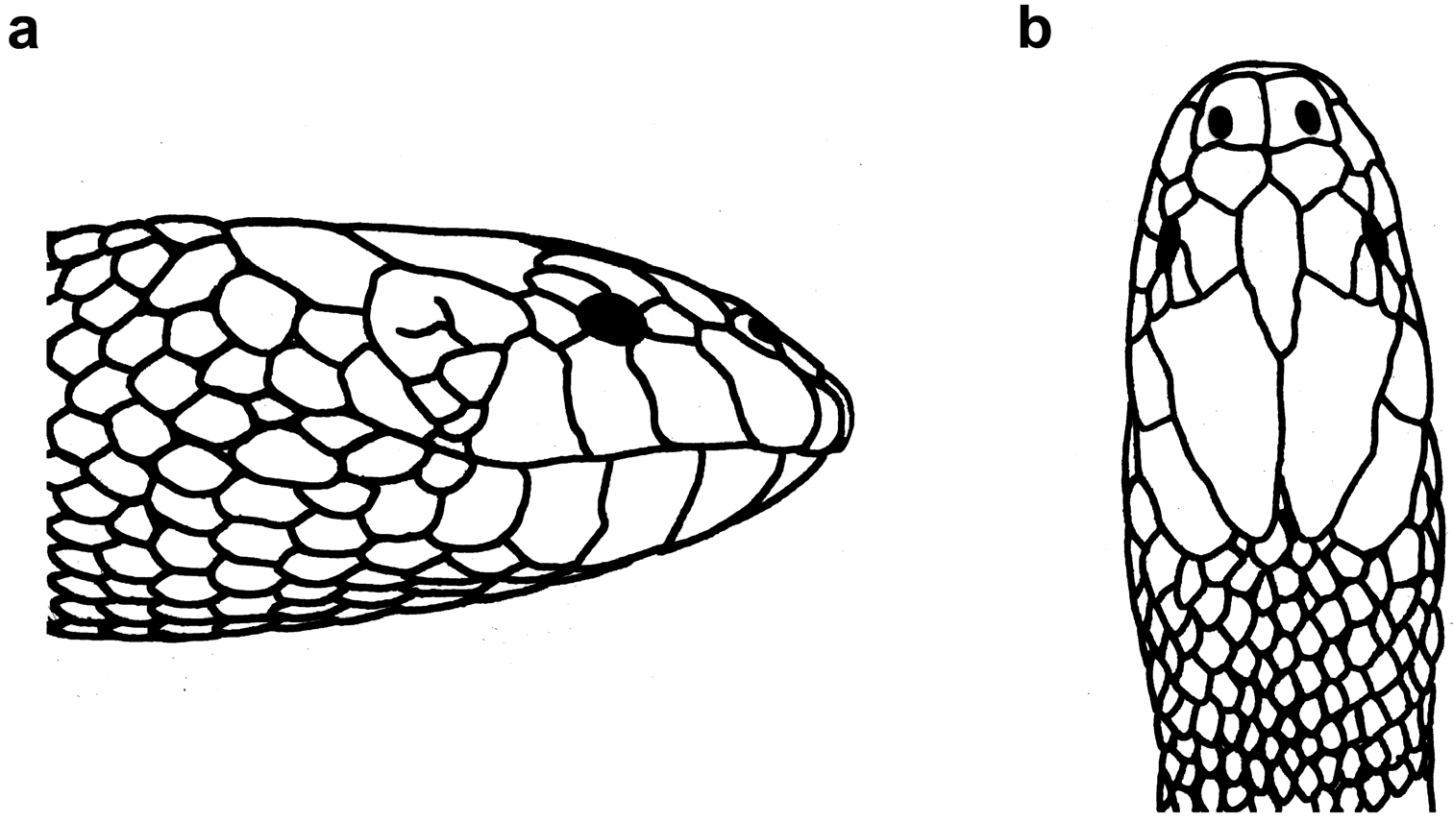
Head of *Hydrophis spiralis*: **a** lateral view **b** dorsal view.

**Figure 15.**
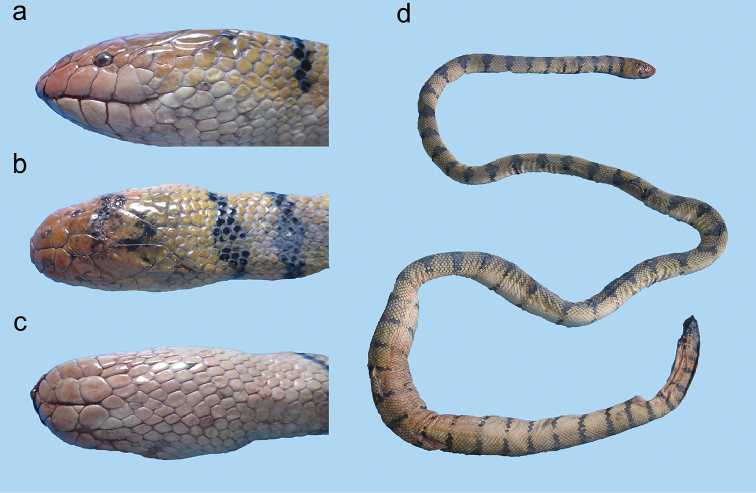
*Hydrophis spiralis*: **a** lateral view **b** dorsal view, and **c** ventral view of head **d** body.

**Figure 16.**
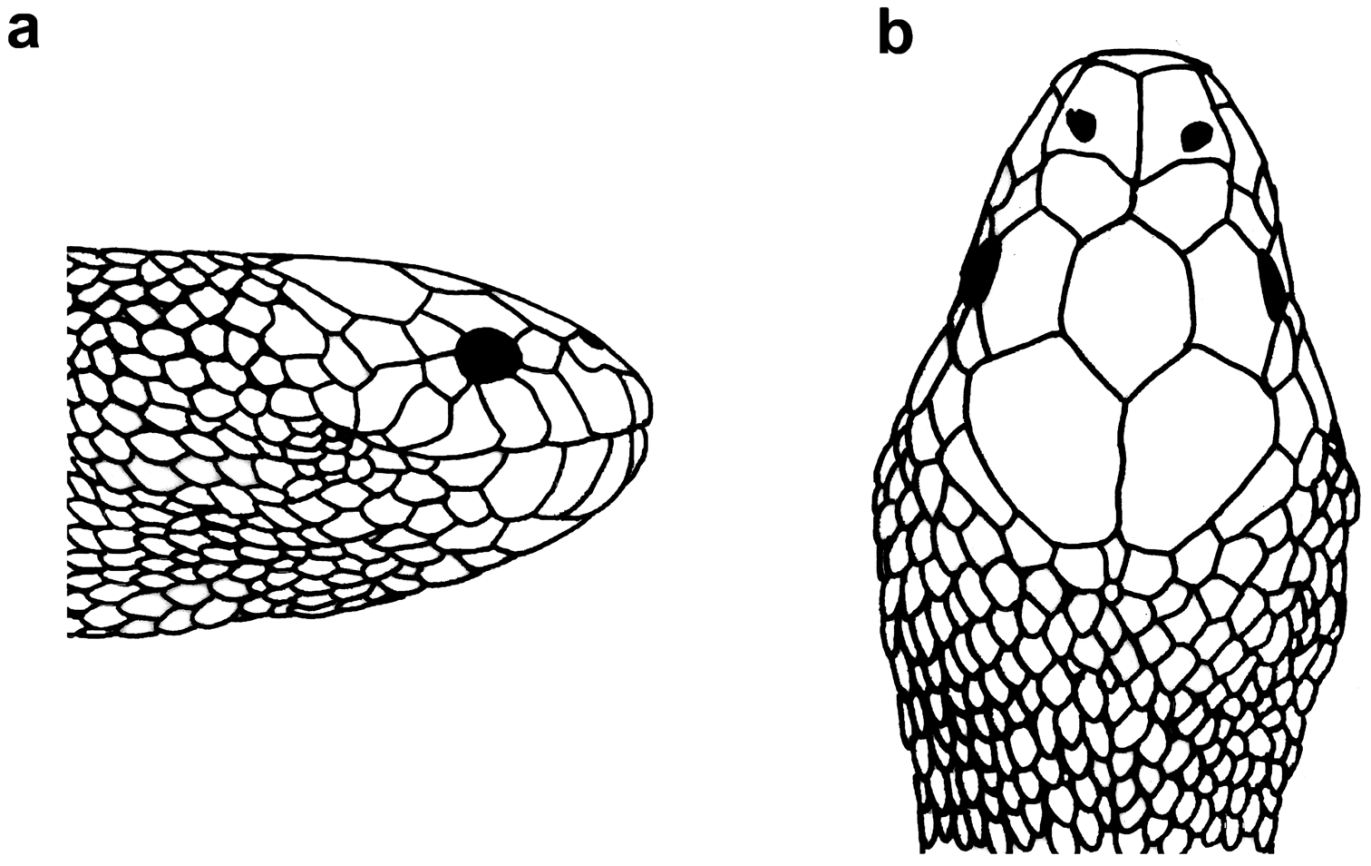
Head of *Hydrophis ornatus*: **a** lateral view **b** dorsal view.

**Figure 17.**
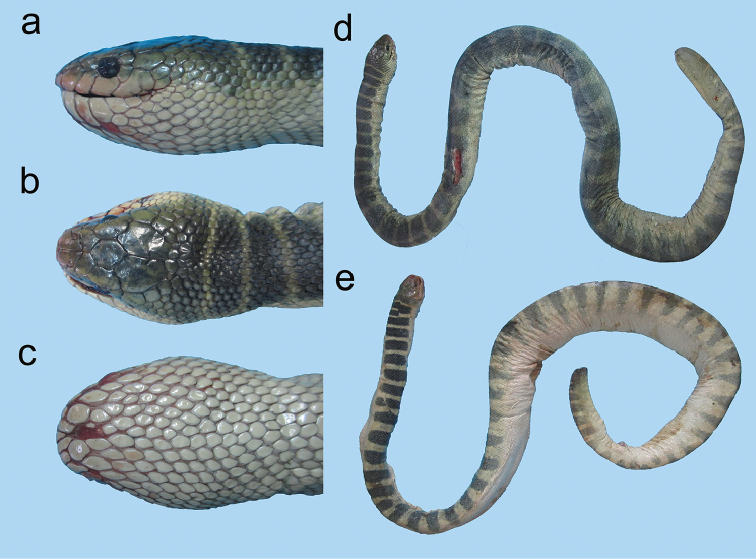
*Hydrophis ornatus*: **a** lateral view **b** dorsal view, and **c** ventral view of head **d** gray body **e** dirty white body.

**Figure 18.**
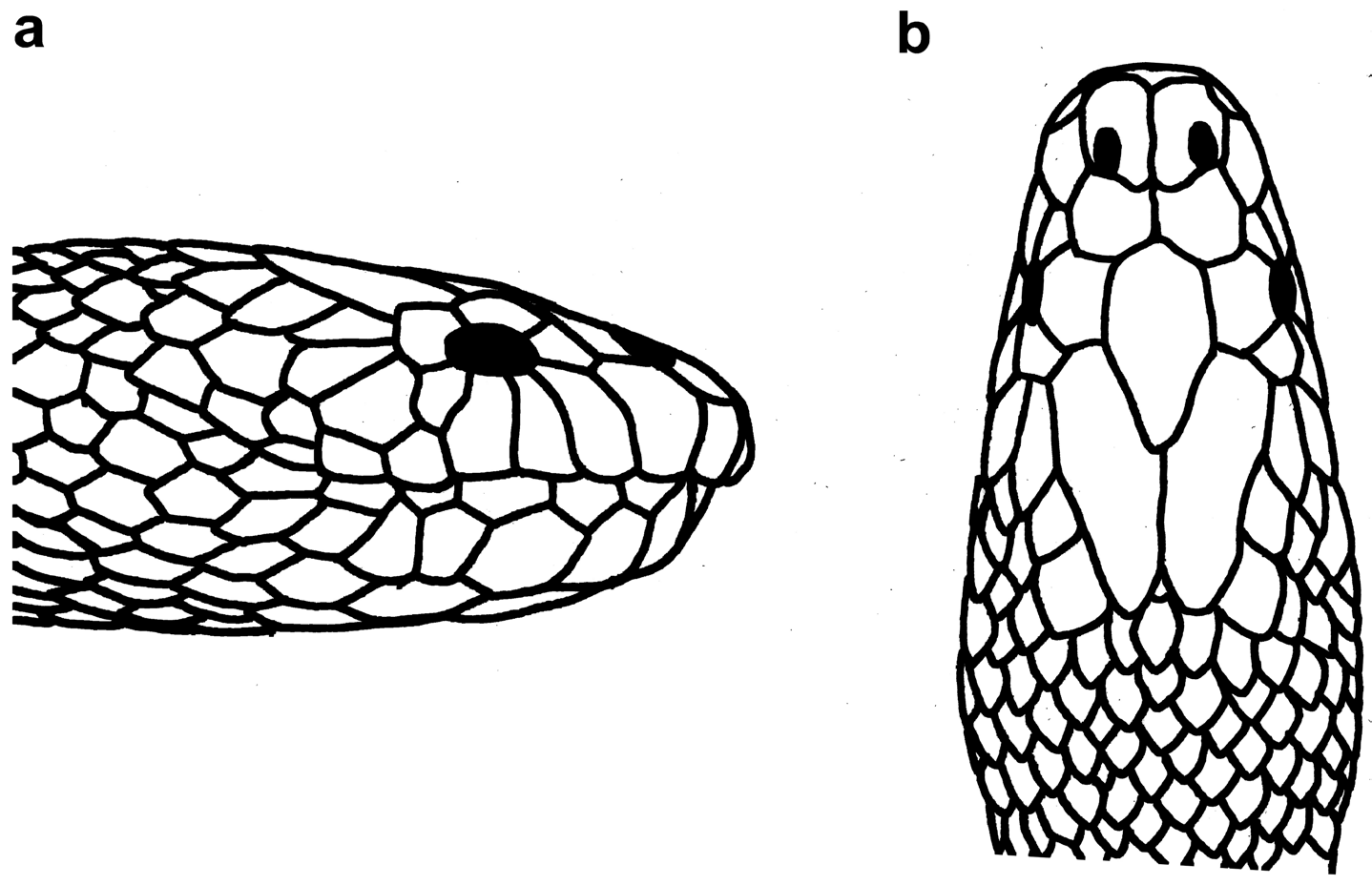
Head of *Hydrophis cyanocinctus*: **a** lateral view **b** dorsal view.

**Figure 19.**
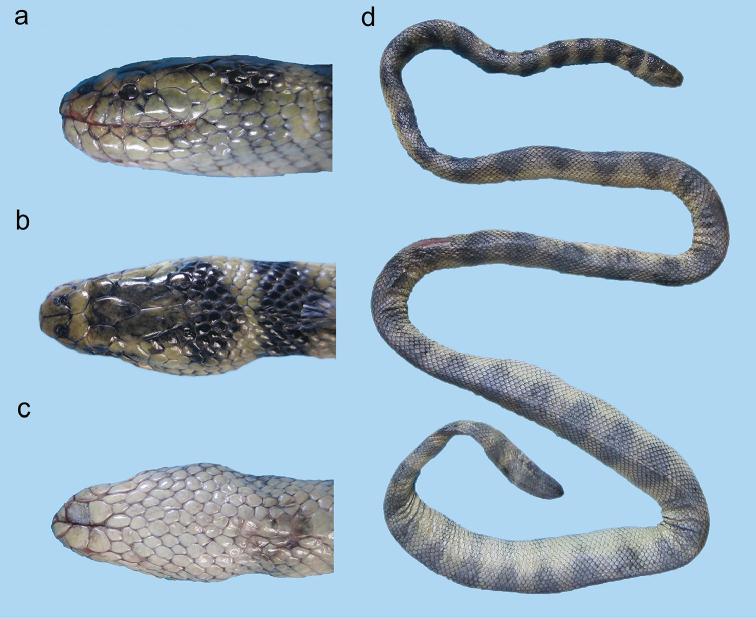
*Hydrophis cyanocinctus*: **a** lateral view **b** dorsal view, and **c** ventral view of head **d** body.

**Figure 20.**
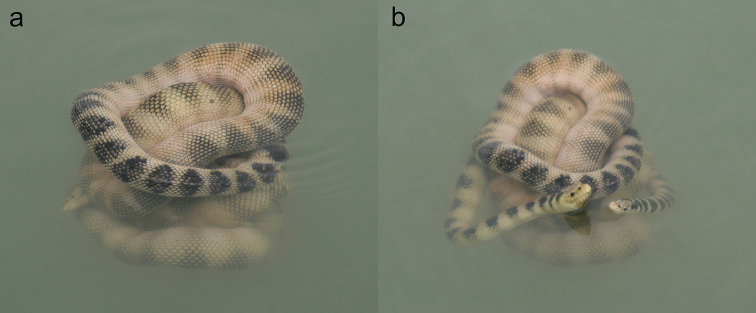
Two *Hydrophis cyanocinctus* intertwined each other and floating on the surface in a mangrove channel in Jask (western Gulf of Oman).

**Figure 21.**
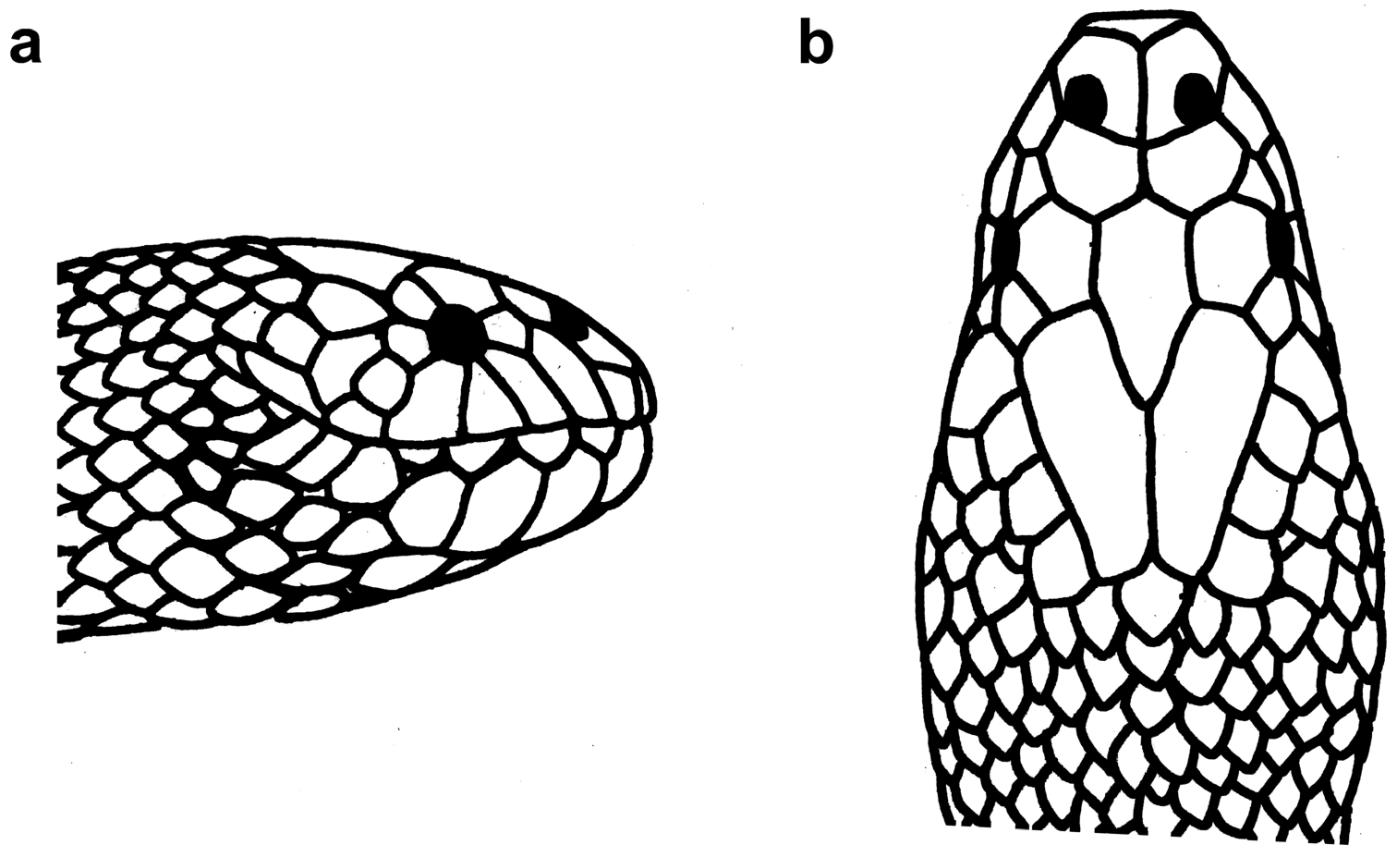
Head of *Hydrophis lapemoides*: **a** lateral view **b** dorsal view.

**Figure 22.**
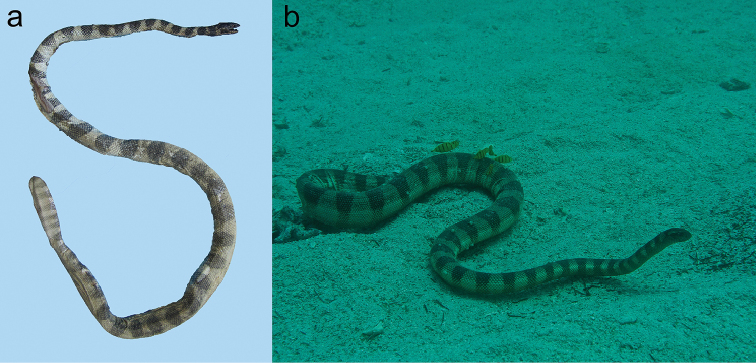
*Hydrophis lapemoides*: **a** body of a specimen examined in this study **b** living individual in coastal waters of Abu Dhabi, UAE, eastern Persian Gulf, depth 10 m, June 2015 (photographed by Rima W. Jabado).

### Key to the species of the genus *Microcephalophis* in the Persian Gulf and Gulf of Oman

**Table:** 

1	Less than 270 ventrals; prefrontal scale usually in contact with second supralabial (Figure 23) (Figures 23–24)	***Microcephalophis gracilis***
–	More than 400 ventrals; prefrontal scale usually in contact with third supralabial (Figure 25) (Figures 25–26)	***Microcephalophis cantoris***

**Figure 23.**
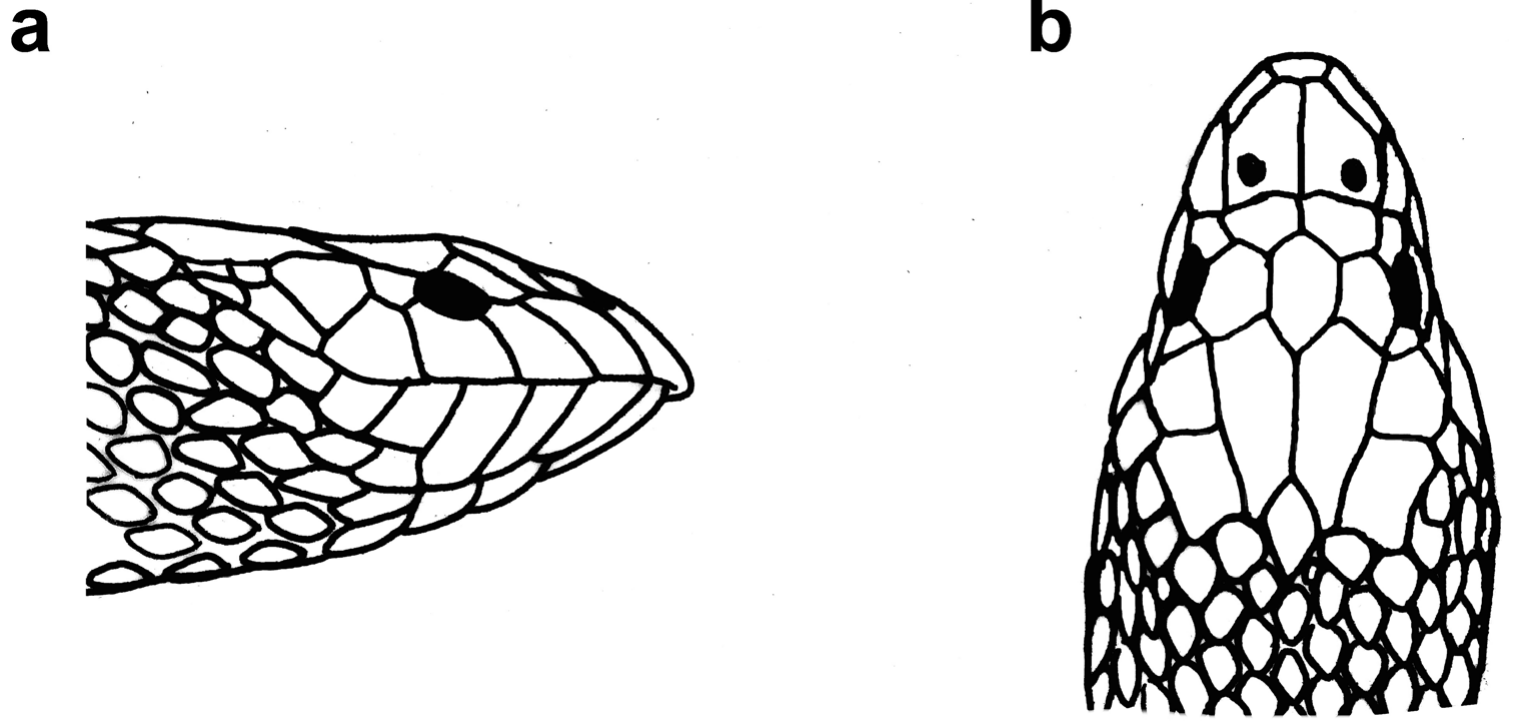
Head of *Microcephalophis gracilis*: **a** lateral view **b** dorsal view.

**Figure 24.**
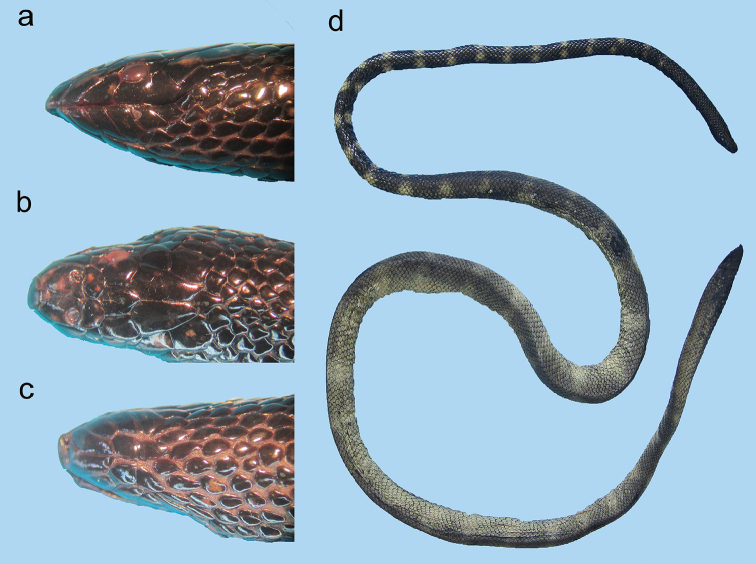
*Microcephalophis gracilis*: **a** lateral view **b** dorsal view, and **c** ventral view of head **d** body.

**Figure 25.**
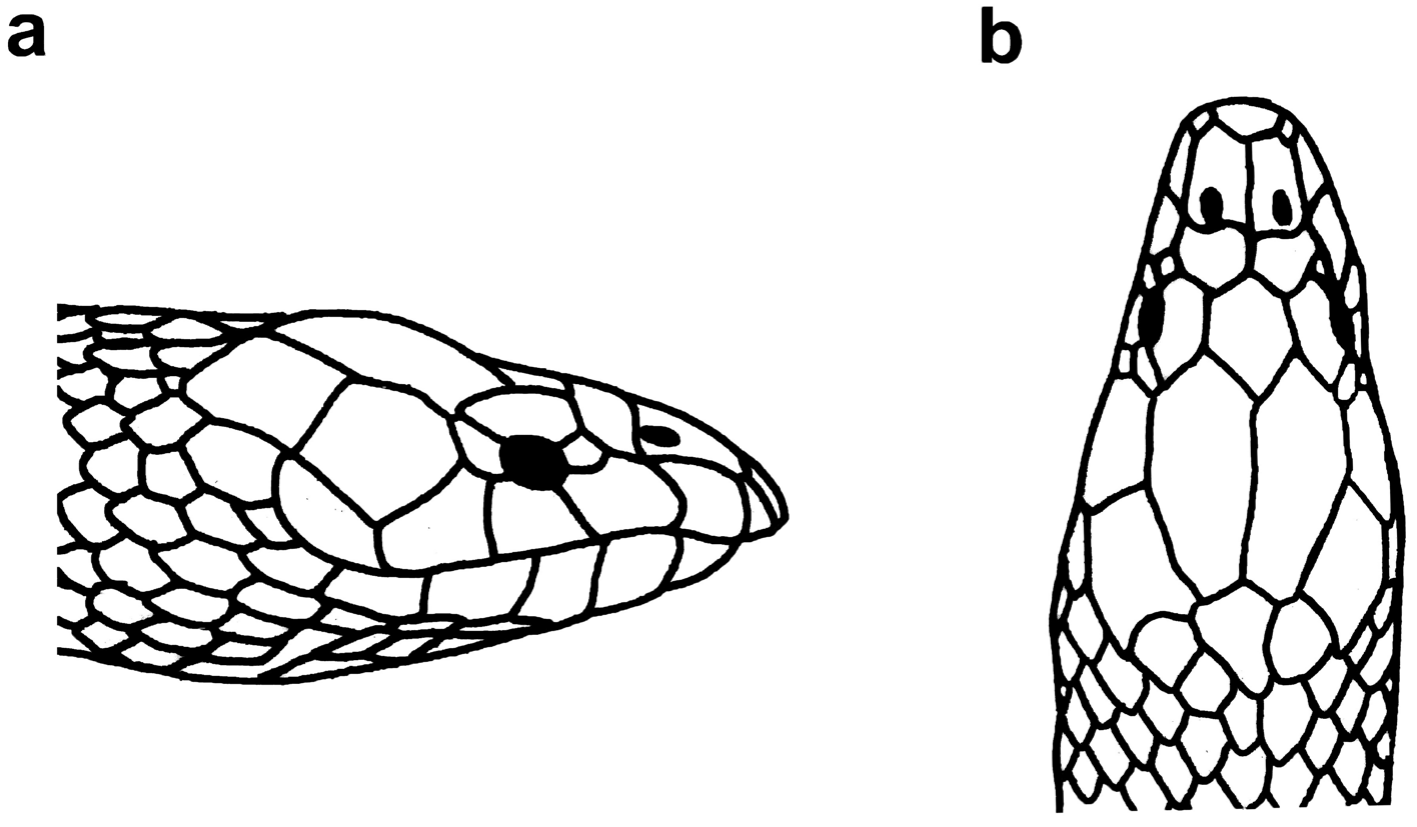
Head of *Microcephalophis cantoris*: **a** lateral view **b** dorsal view.

**Figure 26.**
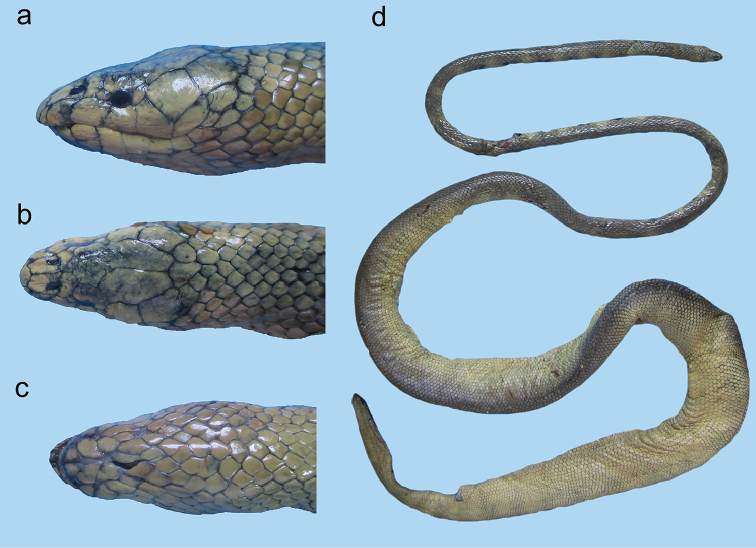
*Microcephalophis cantoris*: **a** lateral view **b** dorsal view, and **c** ventral view of head **d** body.

### Taxonomic accounts Family Elapidae Boie, 1827 Subfamily Hydrophiinae Fitzinger, 1843

#### *Hydrophis* Latreille *in* Sonnini & Latreille, 1801

##### Hydrophis schistosus

Taxon classificationAnimaliaSquamataElapidae

Daudin, 1803

Common names: English – Beaked Sea Snake; Farsi – Mâr-e daryâï-ye nokdâr

Figures 3a , 4e , 5 , 6 , 7

Hydrophis schistosus Daudin, 1803: 386.
Enhydrina schistosa -Boulenger 1887: 408. -Boulenger 1896: 302. -Smith 1926: 39. -Volsøe 1939: 14. -Gasperetti 1988: 306. -Leviton et al. 1992: 120. -Carpenter et al. 1997: 246. -Firouz 1999: 191. -Latifi 2000: 336. -Baldwin and Gardner 2005: 248. -Firouz 2005: 209. -Soorae et al. 2010: 535. -Egan 2007: 143. -Safaei and Esmaili 2009: 45. -Rastegar-Pouyani et al. 2008: 20.
Hydrophis schistosus -Kamali 2013: 242. -Safaei-Mahroo et al. 2015: 282.

###### Material examined.

**Persian Gulf**: 1 specimen, Larak Island [(ZMSBUK.HD.58), TL 971, SVL 857, HL 29.6, HW 12.6, GL 18.4, SNL 5.3, NEL 3.1, ND 52, GBD 100, NSL 7, NSR 47, BSR 57, NV 301], February 2014, collector: M. Ghavasi.

**Gulf of Oman**: 8 specimens, Jask and Ras-Meydani, depth 18–50m [(ZMSBUK.HD.14), TL 1147, SVL 1008, HL 29.4, HW 14.5, GL 20.8, SNL 5.3, NEL 3.7, ND 55, GBD 95, NSL 8, NSR 48, BSR 55, NV 315; (ZMSBUK.HD.21), TL 1180, SVL 1064, HL 31.2, HW 12.3, GL 22.4, SNL 5.4, NEL 3.4, ND 54, GBD 109, NSL 9, NSR 51, BSR 57, NV 303; (ZMSBUK.HD.25), juvenile, TL 508, SVL 451, HL 17.9, HW 7.7, GL 12, SNL 2.9, NEL 1.3, ND 26, GBD 39, NSL 8, NSR 51, BSR 59, NV 344; (ZMSBUK.HD.41), TL 1093, SVL 977, HL 30.5, ND 51, GBD 95, NSL 8, BSR 60; (ZMSBUK.HD.45), TL 1057, SVL 943, HL 31.3, HW 18, GL 20.5, SNL 4.4, NEL 3.9, ND 48, GBD 96, NSL 7, NSR 47, BSR 60, NV 323; (ZMSBUK.HD.50), TL 1172, SVL 1064, HL 36.7, HW 19.3, GL 23.7, SNL 5.5, NEL 4.6, ND 60, GBD 105, NSL 8, NSR 51, BSR 61, NV 316; (ZMSBUK.HD.51), TL 932, SVL 823, HL 28.4, HW 13.7, GL 17.7, SNL 5.6, NEL 3.4, ND 45, GBD 98, NSL 8, NSR 50, BSR 61, NV 325; (ZMSBUK.HD.52), TL 1230, SVL 1093, HL 35.2, HW 16.6, GL 23.5, SNL 4.8, NEL 4.4, ND 62, GBD 116, NSL 8, NSR 53, BSR 65, NV 340], October and November 2013, collector: M. Rezaie-Atagholipour; 3 specimens, Jask, mangrove swamps, depth 1–3m [(ZMSBUK.HD.27), TL 966, SVL 855, HL 28.3, HW12.3, GL 19.7, SNL 4.9, NEL 3.7, ND 44, GBD 90, NSL9, NSR 52, BSR 59, NV 327; (ZMSBUK.HD.29), TL 1080, SVL 947, HL 30.5, HW 18.1, GL 17, SNL 5.6, NEL 4.1, ND 50, GBD 80, NSL 9, NSR 40, BSR 58, NV 348; (ZMSBUK.HD.62), TL 1032, SVL 907, HL 28.5, HW 16, GL 20.1, SNL 5.2, NEL 4.5, ND 52, GBD 88, NSL 8, NSR 47, BSR 59, NV 327], December 2013, collector: M. Rezaie-Atagholipour; 2 specimens, Beris and Pasa-Bandar, depth 20–40m [(ZMSBUK.HD.49), TL 1044, SVL 928, HL 30.8, HW 12.7, GL 19.3, SNL 5, NEL 3.7, ND 50, GBD 105, NSL 7, NSR 51, BSR 64, NV 302; (ZMSBUK.HD.53), TL 1095, SVL 955, HL 31.9, HW 14.7, GL 19.4, SNL 2.5, NEL 4.2, ND 50, GBD 95, NSL 8, NSR, BSR, NV, NB], August 2013, collector: M. Rezaie-Atagholipour.

###### Diagnosis.

Head of medium size; rostral beaked-shaped, elongate with decurved and pointed tip (Figures 2a, 3a); mental elongate, slender and dagger-shaped, hidden in the groove between chin shields (Figures 3a, 6c); body slightly elongate, not markedly slender anteriorly (Figure 6e–f); 301–348 ventrals [340–354 (Volsøe 1939)], small and more or less indistinguishable from adjacent scales at mid-body (Figure 4e); 40–55 scale rows on neck and 53–65 on body [47–52 and 56–60 (Volsøe 1939)].

###### Coloration.

Gray or dark olive dorsally, whitish ventrally; body rings developed in juveniles but paler or absent in adults (Figure 6e); exceptionally black dorsally (Figure 6f).

###### Size.

Mean TL 1036 mm, maximum 1230 mm (n = 14) [n = 3, maximum TL 1350 mm (Volsøe 1939)]

###### General distribution.

Indo- West Pacific, from the Persian Gulf to Australia (David and Ineich 1999).

###### IUCN Red List Category.

Least concern (IUCN 2016).

###### Remarks.

*Hydrophis schistosus* is distinct from other species in the Persian Gulf and Gulf of Oman due to its beaked-shaped rostral and dagger-shaped mental. Results of our field surveys showed that the species is more abundant in the Gulf of Oman rather than in the Persian Gulf. This is supported by other studies as eight specimens have been hitherto examined from the Gulf of Oman (Boulenger 1887, 1896; Smith 1926; Volsøe 1939) versus three specimens from the Persian Gulf (Corkill 1932; Smith 1926; present study). Furthermore, during two months boat surveys in Hara (mangrove) Biosphere Reserve in the eastern Persian Gulf, only two specimens of the species were found (Rezaie-Atagholipour et al. 2012). Nonetheless, two specimens collected by Rezaie-Atagholipour et al. (2012) and the only specimen collected in this study were from Strait of Hormoz in the boundary of the two gulfs. Therefore these three specimens may be vagrant, and can not be strictly allocated to a particular population in the Persian Gulf. Concerning the two remaining specimens reported from the Gulf by Corkill (1932) and Smith (1926), no exact locality was mentioned. In conclusion, populations of *Hydrophis schistosus* in the Persian Gulf seem likely to be in low abundance. On the other hand, results of this study showed that the species is one of the most abundant sea snakes in the Gulf of Oman. In total, 26% of all specimens examined here were *Hydrophis schistosus*, all but one collected from the Gulf of Oman. Approximately 71% (261 of 367 specimens) of sea snakes that Safaei and Esmaili (2009) collected from coastal waters of Hormozgan Province (eastern Persian Gulf and western Gulf of Oman) were *Hydrophis schistosus*. They mentioned that most of these specimens were collected from Gulf of Oman. They failed however to report the exact proportions. Mating behavior of the species was sighted during a boat survey through coastal waters of Jask (western Gulf of Oman) in December 2013, the two animals having been seen intertwined and floating on the surface (Figure 7). *Hydrophis schistosus* is an aggressive sea snake. This snake is prey-specific, mostly consuming spiny catfishes (Glodek and Voris 1982; Voris and Moffett 1981). Recent morphological and molecular evidence revealed that this species consists of two convergent lineages through its geographical distribution range. Now, the Australian lineage is elevated to species status and provisionally referred as to *Hydrophis zweifeli* (see Ukuwela et al. 2013).

##### Hydrophis viperinus

Taxon classificationAnimaliaSquamataElapidae

(Schmidt, 1852)

Common names: English – Viperine Sea Snake; Farsi – Mâr-e daryâï-ye af’ ïshekl

Figures 3b , 4a , 8 , 9

Thalassophis viperina Schmidt, 1852: 79.
Thalassophis viperina -Smith 1926: 35. -Volsøe 1939: 10.
Hydrophis jayakari -Boulenger 1887: 408.
Hydrophis plumbea -Murray 1887: 34.
Distira viperina -Boulenger 1896: 298.
Praescutata viperina -Corkill and Cochrane 1965: 494. -Joger 1984: 31. -Leviton and Aldrich 1984: XXIV. -Gasperetti 1988: 325. -Leviton et al. 1992: 127. -Carpenter et al. 1997: 249. Firouz 1999: 192. -Baldwin and Gardner 2005: 251. -Firouz 2005: 210. -Egan 2007: 166. -Rastegar-Pouyani et al. 2008: 20. -Soorae et al. 2010: 535.
Hydrophis viperinus -Kamali 2013: 244. -Safaei-Mahroo et al. 2015: 282.

###### Material examined.

**Gulf of Oman**: 2 specimens, Beris and Pasa-Bandar, depth 20–40m [(ZMSBUK.HD.20), TL 737, SVL 656, HL 19.1, HW 12.6, GL 14.8, SNL 3, NEL 3, ND 35, GBD 67, NSL 8, NSR 29, BSR 47, NV 250; (ZMSBUK.HD.43), TL 740, SVL 657, HL 18.9, HW 14.9, GL 13.3, SNL 3.9, NEL 2.8, ND 38, GBD 68, NSL 7, NSR 30, BSR 43, NV 265], August 2013, collector: M. Rezaie-Atagholipour.

###### Diagnosis.

Head large, short and depressed (Figures 8–9); tip of rostral curved and markedly tridentate (Figures 2b, 3b); usually 7–8 supralabials, none in contact with prefrontal (Figure 8); 250–265 large ventrals [245–291 (Volsøe 1939)], markedly distinguishable from adjacent scales (Figure 4a), larger anteriorly than posteriorly; ventrals on anterior part of body wide and enlarged, half width of body, more or less rectangular in shape; 29–38 scale rows on neck, 39–47 on body [27–31 and 40–43 (Volsøe 1939)].

###### Coloration.

Dark gray dorsally, dirty white ventrally; with or without pale body bands broadest dorsally; tip of tail usually black (Figure 9d).

###### Size.

Maximum TL 740 mm (n = 2); [n = 8, mean TL 662 mm, maximum TL 780 mm (Volsøe 1939)].

###### General distribution.

Indian Ocean, from the Persian Gulf to Malay Archipelago (David and Ineich 1999).

###### IUCN Red List Category.

Least concern (IUCN 2016).

###### Remarks.

*Hydrophis viperinus* is distinct from other sea snakes in the Persian Gulf and Gulf of Oman by having markedly rectangular-shaped and enlarged ventrals on the anterior part of the body. Smith (1926) and Wall (1921) mentioned that the Persian Gulf is the westernmost distribution limit of *Hydrophis viperinus*. Volsøe (1939) had doubts about it: “their only exact records from the Persian Gulf are however from Muscat, which is situated about 400 km outside the Strait of Hormoz”. We agree with Volsøe (1939) as the two specimens we examined in this study and the specimens examined in other studies in the area (e.g. Boulenger 1887; Volsøe 1939) have all been collected from the Gulf of Oman. We did not find any specimen of *Hydrophis viperinus* in Iranian coastal waters of the Persian Gulf during our field surveys. Therefore, population of *Hydrophis viperinus* in the Persian Gulf, if present, seems likely to be in low abundance.

##### Hydrophis curtus

Taxon classificationAnimaliaSquamataElapidae

(Shaw, 1802)

Common names: English – Shaw’s Sea Snake, Short Sea Snake, Spine-bellied Sea Snake; Farsi – Mâr-e daryâï-ye kutâh

Figures 3c , 4b , 10 , 11

Hydrus curtus Shaw, 1802: 562.
Enhydris curtus -Werner 1895: 19.
Lapemis curtus -Smith 1926: 112. -Kennedy 1937: 748. -Volsøe 1939: 21. -Corkill and Cochrane 1965: 495. -Joger 1984: 29. -Leviton and Aldrich 1984: XXIV. -Gasperetti 1988: 317. -Leviton et al. 1992: 125. -Carpenter et al. 1997: 248. -Firouz 1999: 192. -Baldwin and Gardner 2005: 250. -Firouz 2005: 210. -Soorae et al. 2006: 109. -Rastegar-Pouyani et al. 2008: 20. -Safaei and Esmaili 2009: 45. -Soorae et al. 2010: 535. -Kordi and Shabanipour 2012: 71. -Rezaie-Atagholipour 2012: 494. - Sereshk and Bakhtiari 2014: 116. -Sereshk and Bakhtiari 2015: 15781.
Hydrophis curtus -Kamali 2013: 237. -Safaei-Mahroo et al. 2015: 282.

###### Material examined.

**Persian Gulf**: 8 specimens, Bushehr Province [(ZMSBUK.HD.1), TL 915, SVL 831, HL 32.9, HW 24.9, GL 22.9, SNL 7.9, NEL 4.8, ND 74, GBD 130, NSL 8, NSR 32, BSR 39, NV 158, NB 39; (ZMSBUK.HD.2), TL 900, SVL 805, HL 34, HW 20.5, GL 23, SNL 7.3, NEL 5.4, ND 80, GBD 130, NSL 8, NSR 29, BSR 37, NV 187, NB 45; (ZMSBUK.HD.5), TL 835, SVL 755, HL 31.5, HW 16.8, GL 26, SNL 7.1, NEL 4.6, ND 62, GBD 95, NSL 8, NSR 30, BSR 39, NV 165, NB 47; (ZMSBUK.HD.11), TL 852, SVL 751, HL 30.3, HW 16.8, GL 22.2, SNL 5.8, NEL 4.5, ND 72, GBD 107, NSL 9, NSR 28, BSR 33, NV 147, NB 50; (ZMSBUK.HD.15), TL 1008, SVL 910, HL 34.2, HW 19.2, GL 23.9, SNL 6.6, NEL 5, ND 80, GBD 125, NSL 8, NSR 31, BSR 39, NV 185, NB 55; (ZMSBUK.HD.17), TL 869, SVL 781, HL 28.7, HW 15.8, GL 20.7, SNL 6, NEL 4.3, ND 61, GBD 75, NSL 7, NSR 24, BSR 36, NV 153, NB 48; (ZMSBUK.HD.47), TL 797, SVL 716, HL 30.5, HW 16.9, GL 19.7, SNL 5.6, NEL 4.6, ND 50, GBD 62, NSL 8, NSR 30, BSR 37, NV 158, NB 45; (ZMSBUK.HD.48), TL 716, SVL 642, HL 28.4, HW 14.8, GL 18.4, SNL 4.3, NEL 4.2, ND 55, GBD 85, NSL 8, NSR 33, BSR 43, NV 199, NB 46], September 2013, collector: M. Rezaie-Atagholipour; 1 specimen, Larak Island [(ZMSBUK.HD.61), TL 783, SVL 700, HL 28.5, HW 15.3, GL 20.3, SNL 5, NEL 4.7, ND 62, GBD 95, NSL 8, NSR 32, BSR 42, NV 200, NB 51], February 2014, collector: M. Ghavasi.

**Gulf of Oman**: 6 specimens, Beris and Pasa-Bandar, depth 20–40m [(ZMSBUK.HD.8), TL 855, SVL 772, HL 34.1, HW 21.6, GL 21.5, SNL 5.43, NEL 4.91, ND 70, GBD 93, NSL 7, NSR 28, BSR 34, NV 158; (ZMSBUK.HD.18), TL 960, SVL 865, HL 34, HW 16.5, GL 25, SNL 5.8, NEL 5.7, ND 72, GBD 115, NSL 7, NSR 32, BSR 37, NV 157; (ZMSBUK.HD.19), TL 825, SVL 753, HL 28.9, HW 18.1, GL 19.2, SNL 4.8, NEL 4.5, ND 58, GBD 100, NSL 8, NSR 33, BSR 40, NV201, NB 47; (ZMSBUK.HD.44), TL 1015, SVL 925, HL 40.9, HW 27.7, GL 25.6, SNL 6.6, NEL 5.3, ND 73, GBD 99, NSL 8, NSR 34, BSR 43, NV 197, NB 49; (ZMSBUK.HD.57), TL 745, SVL 674, HL 29.7, HW 17.6, GL 20.3, SNL 3.9, NEL 3.6, ND 57, GBD 85, NSL 7, NSR 33, BSR 38, NV 185, NB 50; (ZMSBUK.HD.60), TL 965, SVL 872, HL 32.7, HW 18.1, GL 23.4, SNL 5.6, NEL 5.5, ND 65, GBD 117, NSL 8, NSR31, BSR 39, NV 174, NB 51], August 2013, collector: M. Rezaie-Atagholipour.

###### Diagnosis.

Head large (Figure 11); tip of rostral markedly tridentate (Figures 2c, 3c); parietals divided into small shields (Figures 10b, 11d–f); 7–9 supralabials, second contacts with prefrontal (Figure 10), third and fourth or only fourth touch eye; body short and stout (Figure 11j–l); 147–201 medium size ventrals, distinguishable from the adjacent scales (Figure 4b), larger anteriorly than posteriorly; ventrals on anterior part of body more or less hexagonal in shape, less than half width of body; 28–38 scale rows on neck and 32–43 on body [27–31 and 32–38 (Volsøe 1939)].

###### Coloration.

Three color patterns observed in this study: 1- usually gray dorsally, gray-whitish ventrally with pale gray dorsal bands (Figures 11a, d, g, j); 2- rarely yellowish body with blackish dorsal bands (Figure 11c, f, i, l); 3- rarely black-grayish body with black dorsal bands (Figure 11b, e, h, k); 39–55 dorsal bands in all three types usually fused laterally, zigzag in form; tip of tail usually black.

###### Size.

Mean TL 716 mm, maximum 1015 mm (n = 15); [n = 12, mean TL 606 mm, maximum TL 860 mm (Volsøe 1939)].

###### General distribution.

Indo- West Pacific, from the Persian Gulf to Australia (David and Ineich 1999).

###### IUCN Red List Category.

Least concern (IUCN 2016).

###### Remarks.

In the Persian Gulf and Gulf of Oman, *Hydrophis curtus* may be roughly confused with *Hydrophis ornatus* at a glance. *Hydrophis curtus* however can be quickly distinguished from *Hydrophis ornatus* by having parietals divided into small shields and laterally fused dorsal bands, zigzag in form, versus clearly distinguishable dorsal bands in *Hydrophis ornatus*. Volsøe (1939) mentioned that *Hydrophis curtus* is one of the most abundant sea snakes in the Iranian coastal waters of the eastern Persian Gulf. Our results show that it is abundant in the western part of the Iranian Persian Gulf (Bushehr Province) as well. *Hydrophis curtus* is a diet generalist, known to prey on fish belonging to 33 families, cuttlefish and amphipods (Rezaie-Atagholipour 2012).

##### Hydrophis platurus

Taxon classificationAnimaliaSquamataElapidae

(Linnaeus, 1766)

Common names: English – Pelagic Sea Snake, Yellow-bellied Sea Snake; Farsi – Mâr-e daryâï-ye shekam zard

Figures 3h , 4f , 12 , 13

Anguis platura Linnaeus, 1766: 391.
Hydrus platurus -Werner 1895: 18. -Boulenger 1897: 468.
Pelamis platurus -Volsøe 1939: 23. -Corkill and Cochrane 1965: 495. -Leviton and Anderson 1967: 188. -Gallagher 1971: 31. -Eissa and El-Assy 1975: 129. -Joger 1984: 30. -Leviton and Aldrich 1984: XXIV. -Gasperetti 1988: 323. -Leviton et al. 1992: 126. -Carpenter et al. 1997: 248. -Firouz 1999: 192. -Latifi 2000: 347. -Baldwin and Gardner 2005: 251. -Firouz 2005: 210. -Soorae et al. 2006: 109. -Egan 2007: 163. -Rastegar-Pouyani et al. 2008: 20. -Safaei and Esmaili 2009: 45. -Soorae et al. 2010: 535.
Hydrophis platurus -Kamali 2013: 241. -Safaei-Mahroo et al. 2015: 282.

###### Material examined.

**Gulf of Oman**: 1 specimen, Jask and Ras-Meydani , depth 18–50m [(ZMSBUK.HD.26), TL 401, SVL 352, HL 25, HW 10.1, GL 17.7, SNL 3.4, NEL 4.7, ND 34, GBD 38, NSL 10, NSR 39, BSR 46], October and November 2013, collector: M. Rezaie-Atagholipour; 2 specimens, Beris and Pasa-Bandar, depth 20–40m [(ZMSBUK.HD.12), TL 611, SVL 551, HL 34.5, HW 13, GL 22.2, SNL 4.7, NEL 6, ND 27, GBD 63, NSL 9, NV 333; (ZMSBUK.HD.23), TL 690, SVL 618, HL 35.8, HW 17.9, GL 29.7, SNL 5, NEL 6.8, ND 52, GBD 72, NSL 9, NSR 56, BSR 58, NV 330], August 2013, collector: M. Rezaie-Atagholipour.

###### Diagnosis.

Head narrow; snout elongate (Figures 12–13); 9–10 supralabials, second touches prefrontal scale, four and fifth separated from eye by suboculars or contact eye (Figures 12a, 13a); body short, not stout (Figure 13d); 330–333 small ventral scales [265–367 (Volsøe 1939)], more or less indistinguishable from adjacent scales at mid-body (Figure 4f).

###### Coloration.

This species has a unique color pattern making it distinguishable from other sea snakes; dorsal half of head and body black, dark green or dark brown, ventral half of head and body yellow, a markedly sharp contrast between dorsal and ventral portions; tail yellow in ventral portion, spotted or barred in dorsal portion (Figure 13d); sometimes with pale dorsal color.

###### Size.

Mean TL 567 mm, maximum 690 mm (n = 3); [n = 5, mean TL 449 mm, maximum 565 mm (Volsøe 1939)].

###### General distribution.

Indo-Pacific, from east and south of Africa to the west coast of Americas (Heatwole 1999).

###### IUCN Red List Category.

Least concern (IUCN 2016).

###### Remarks.

*Hydrophis platurus* is distinguishable from other species in the region by having a unique color pattern (see above). *Hydrophis platurus* has been known as the only planktonic tetrapod, spending a considerable portion of its life floating at the depth of 20–50 m, but preying on fish at the sea surface by float-and-wait feeding strategy, passively drifting with surface and subsurface marine currents. It has consequently the widest distribution of all squamatan reptiles (Cook and Brischoux 2014; Hecht et al. 1974; Sheehy III et al. 2012). None of the specimens collected in this project were from the Persian Gulf, although there are records from the Persian Gulf (e.g. Eissa and El-Assy 1975; Gallagher 1971; Soorae et al. 2006; Volsøe 1939; Werner 1895).

##### Hydrophis spiralis

Taxon classificationAnimaliaSquamataElapidae

(Shaw, 1802)

Common names: English – Yellow Sea Snake; Farsi – Mâr-e daryâï-ye zard

Figures 3f , 14 , 15

Hydrus spiralis Shaw, 1802: 564.
Hydrus temporalis -Blanford 1881: 680.
Hydrus robusta -Boulenger 1887: 408.
Hydrophis spiralis -Smith 1926: 48. -Volsøe 1939: 15. -Haas 1961: 21. -Leviton et al. 1992: 124. -Carpenter et al. 1997: 247. -Firouz 1999: 192. -Latifi 2000: 343. -Baldwin and Gardner 2005: 250. -Rastegar-Pouyani et al. 2008: 20. -Safaei and Esmaili 2009: 45. -Kamali 2013: 243. -Safaei-Mahroo et al. 2015: 282.
Hydrophis spiralis spiralis -Corkill and Cochrane 1965: 495. -Joger 1984: 34. -Leviton and Aldrich 1984: XXIV. -Gasperetti 1988: 315. -Firouz 2005: 209. -Egan 2007: 154. -Soorae et al. 2010: 535.

###### Material examined.

**Gulf of Oman**: 1 specimen, Jask, depth 1–3m [(ZMSBUK.HD.55), TL 1925, SVL 1775, HL 44.3, HW 20.6, GL 28, SNL 7.5, NEL 5.8, ND 67, GBD 90, NSL 7, NSR 30, BSR 38, NV 387, NB 61], December 2013, collector: M. Rezaie-Atagholipour.

###### Diagnosis.

Head of medium size (Figure 15); second supralabial touches prefrontal scale (Figure 14); body markedly elongate, not slender anteriorly (Figure 15d); 387 ventrals [363–385 (Volsøe 1939)], slightly distinguishable from adjacent scales; [27–31 scale rows on neck, 34–38 on body (n = 4, Volsøe 1939; n = 1, present study)].

###### Coloration.

Yellowish body with 61 [30–60 more or less (Gasperetti 1988)] narrow black rings, narrower than yellowish interspaces; head yellowish as body (Figure 15); [a black ventral line sometimes present, head blackish with a horseshoe-shaped mark above in young individuals (Gasperetti 1988)].

###### Size.

TL 1925 mm (n = 1); [n = 4, mean TL 1587 mm; maximum TL 1984 mm (Volsøe 1939)].

###### General distribution.

Indian Ocean, from the Persian Gulf to Malay Archipelago (David and Ineich 1999).

###### IUCN Red List Category.

Least concern (IUCN 2016).

###### Remarks.

*Hydrophis spiralis* is distinguishable from other species in the area by its yellow body and narrow black rings (narrower than yellow interspaces). This species is the longest among all marine hydrophiines (Heatwole 1999). We could catch only one specimen of *Hydrophis spiralis*, which was collected from Jask in the western Gulf of Oman. Other authors however recorded the species from the Persian Gulf (e.g. Blanford 1881; Haas 1961; Volsøe 1939).

##### Hydrophis ornatus

Taxon classificationAnimaliaSquamataElapidae

(Gray, 1842)

Common names: English – Ornate Reef Sea Snake, Ornate Sea Snake; Farsi – Mâr-e daryâï-ye ârâsteh

Figures 3d , 3e , 4c , 16 , 17

Aturia ornata Gray, 1842: 61.
Hydrophis elliotti -Boulenger 1887: 408.
Distira ornata -Werner 1895: 19. -Boulenger 1896: 290.
Hydrophis ornatus ornatus -Smith 1926: 81. -Corkill and Cochrane 1965: 494. -Joger 1984: 34. -Leviton and Aldrich 1984: XXIV. -Gasperetti 1988: 315. -Firouz 2005: 209.
Hydrophis ornatus -Volsøe 1939: 18. -Leviton et al. 1992: 123. -Carpenter et al. 1997: 247. -Firouz 1999: 192. -Latifi 2000: 342. -Baldwin and Gardner 2005: 249. -Egan 2007: 151. -Rastegar-Pouyani et al. 2008: 20. -Safaei and Esmaili 2009: 45. -Soorae et al. 2010: 535. -Kamali 2013: 240. -Safaei-Mahroo et al. 2015: 282.

###### Material examined.

**Persian Gulf**: 3 specimens, Bushehr Province [(ZMSBUK.HD.10), TL 813, SVL 722, HL 26, HW 16.7, GL 18.8, SNL 4.9, NEL 3.9, ND 53, GBD 80, NSL 7, NSR 35, BSR 42, NB 41; (ZMSBUK.HD.13), TL 879, SVL 791, HL 28.1, HW 15, GL 12.5, SNL 4.2, NEL 4, ND 55, GBD 89, NSL 7, NSR 40, BSR 44, NV 302, NB 46; (ZMSBUK.HD.16), TL 1200, SVL 1072, HL 36.6, HW 23.7, GL 27.6, SNL 6, NEL 5.2, ND 74, GBD 157, NSL 7, NSR 41, BSR 50, NV 306, NB 53], September 2013, collector: M. Rezaie-Atagholipour.

**Gulf of Oman**: 3 specimens, Beris and Pasa-Bandar, depth 20–40m [(ZMSBUK.HD.3), TL 1015, SVL 908, HL 32.9, HW 30, GL 21.9, SNL 6.9, NEL 5.2, ND 68, GBD 110, NSL 8, NSR 37, BSR 42, NV 251, NB 51; (ZMSBUK.HD.7), TL 985, SVL 800, HL 32.6, HW 19.5, GL 23, SNL 6.5, NEL 5.2, ND 65, GBD 97, NSL 7, NSR 38, BSR 48, NV 260, NB 51; (ZMSBUK.HD.59), TL 1035, HL 33.9, HW 20.1, GL 24.1, SNL 5, NEL 5.3, ND 65, GBD 140, NSL 8, NSR 34, BSR 48, NV 286, NB 49], August 2013, collector: M. Rezaie-Atagholipour.

###### Diagnosis.

Head of medium size (Figure 17); 7–8 supralabials, second usually in contact with prefrontal, third and fourth, or third, fourth and fifth touch eye (Figure 16); body slightly stout, not markedly elongate (Figure 17d–e); 251–306 ventrals, slightly distinguishable from adjacent scales (Figure 4c); 34–43 scale rows on neck and 40–50 on body.

###### Coloration.

Body dirty white (Figure 17e) to grayish (Figure 17d) with 41–53 rhomboidal or rectangular black or dark olive bands along body and tail, clearly distinguishable from each other (Figure 17 d-e).

###### Size.

Mean TL 988 mm, maximum 1200 mm (n = 6); [n = 1, TL 885 mm (Volsøe 1939)].

###### General distribution.

Indo-West Pacific, from the Persian Gulf to Australia (David and Ineich 1999; Smith 1926).

###### IUCN Red List Category.

Least concern (IUCN 2016).

###### Remarks.

In the Persian Gulf and Gulf of Oman, *Hydrophis ornatus* may be roughly misidentified with *Hydrophis curtus* (for more details see remarks of *Hydrophis curtus*).

##### Hydrophis cyanocinctus

Taxon classificationAnimaliaSquamataElapidae

Daudin, 1803

Common names: English – Annulated Sea Snake, Bluebanded Sea Snake; Farsi – Mâr-e daryâï-ye halqehdâr

Figures 3g , 18 , 19 , 20

Hydrophis cyanocinctus Daudin, 1803: 383.
Hydrophis cyanocinctus -Smith 1926: 56. -Schmidt 1939: 87. -Volsøe 1939: 17. -Laurent 1948: 9. -Haas 1957: 87. -Haas 1961: 21. -Corkill and Cochrane 1965: 494. -Leviton and Anderson 1967: 188. -Gallagher 1971: 31. -Eissa and El-Assy 1975: 129. -Joger 1984: 33. -Leviton and Aldrich 1984: XXIV. -Gasperetti 1988: 310. -Leviton et al. 1992: 121. -Carpenter et al. 1997: 246. -Firouz 1999: 192. -Latifi 2000: 338. -Baldwin and Gardner 2005: 248. -Firouz 2005: 209. -Egan 2007: 145. -Rastegar-Pouyani et al. 2008: 20. -Safaei and Esmaili 2009: 45. -Soorae et al. 2010: 535. -Calvete et al. 2012: 4091. -Rezaie-Atagholipour et al. 2012: 53. -Rezaie-Atagholipour et al. 2012b: 416. -Kamali 2013: 238. Rezaie-Atagholipour et al. 2013: 328. -Safaei-Mahroo et al. 2015: 282. -Sereshk and Bakhtiari 2015: 15781. -Khorjestan et al. 2016: 45.
Hydrophis cyanocincta -Boulenger 1887: 408.
Distira cyanocincta -Werner 1895: 19. -Boulenger 1896: 294.

###### Material examined.

**Persian Gulf**: 1 specimen, Bushehr Province [(ZMSBUK.HD.9), TL 1185, SVL 1075, HL 23.9, HW 12.5, GL 13.4, SNL 4, NEL 3.6, ND 44, GBD 67, NSL 8, NSR 28, BSR 41, NV 359, NB 54], September 2013, collector: M. Rezaie-Atagholipour.

**Gulf of Oman**: 3 specimens, Beris and Pasa-Bandar, depth 20–40m [(ZMSBUK.HD.4), TL 1275, SVL 1160, HL 25.3, HW 10.7, GL 13.3, SNL 5, NEL 4.4, ND 38, GBD 82, NSL 8, NSR 29, BSR 39, NV 332, NB 56; (ZMSBUK.HD.6), TL 1447, SVL 1332, HL 27.3, HW 16.8, GL 17.9, SNL 4.6, NEL 3.2, ND 24, GBD 105, NSL 8, NSR 29, BSR 40, NV 339, NB 53; (ZMSBUK.HD.56), TL 1065, SVL 463, HL 14.8, HW 10.5, GL 13.9, SNL 3.7, NEL 2.5, ND 38, GBD 67, NSL 8, NSR 25, BSR 39, NV 300, NB 51], August 2013, collector: M. Rezaie-Atagholipour.

###### Diagnosis.

Head slightly small (Figure 19); 7–8 supralabials, second in contact with prefrontal (Figure 18), third, fourth and fifth [or third and fourth, or fourth and fifth (Gasperetti 1988)] touch eye; body elongate but not markedly slender anteriorly (Figure 19d); 300–359 ventrals [345–372 (Volsøe 1939)], slightly distinguishable from adjacent scales at mid-body; body scales on thickest part of the body with round or bluntly pointed tips, slightly or distinctly imbricate; 25–31 scale rows on neck, 39–41 on body [28–31 and 38–44 (Volsøe 1939)].

###### Coloration.

Body dark olive, grayish, or dirty white, dorsally darker and ventrally paler; 51–56 [44–54 (Volsøe 1939)] black rings broader dorsally, or broader bands tapering to points on laterals, on body and tail (Figure 19d); head black in juveniles, usually with a yellow horseshoe-shaped mark above (Figure 19b); adults with head sometimes of same color as body without the horseshoe-shaped mark (Figure 20b).

###### Size.

Mean TL 1243 mm, maximum 1447 mm (n = 4); [n = 7, mean TL 1195 mm, maximum TL 1495 mm (Volsøe 1939)].

###### General distribution.

Indo-West Pacific, from the Persian Gulf to Japan (David and Ineich 1999).

###### IUCN Red List Category.

Least concern (IUCN 2016).

###### Remarks.

In the Persian Gulf and Gulf of Oman, juveniles of *Hydrophis cyanocinctus* (smaller than one meter) are morphologically close to *Hydrophis lapemoides*. In this case, focusing on the shape of scales in the thickest part of the body (with rounded or bluntly pointed tips versus more or less hexagonal or quadrangular in shape in *Hydrophis lapemoides*) is helpful (see diagnostic features for both species). But adults exceed one meter, which is very rare in *Hydrophis lapemoides*. Wall (1921) mentioned that *Hydrophis cyanocinctus* is probably the most abundant species in the region. Volsøe (1939) however mentioned that in the Persian Gulf it is equaled or even surpassed by *Hydrophis lapemoides* and *Hydrophis curtus*. Gasperetti (1988) mentioned that both *Hydrophis cyanocinctus* and *Hydrophis lapemoides* are the most abundant sea snakes in both gulfs. Rezaie-Atagholipour et al. (2012) mentioned that *Hydrophis cyanocinctus* is the most abundant sea snake in Hara Biosphere Reserve (the largest mangrove stand in the northwestern Indian Ocean) of the Persian Gulf. Intertwining of two *Hydrophis cyanocinctus* is sometimes observed in the mangrove channels of the same ecosystem and other mangrove stands in the area (Figure 20; for more details see Rezaie-Atagholipour et al. 2012). Rezaie-Atagholipour et al. (2013) studied feeding habits of the same population in the biosphere reserve and found that main prey items for *Hydrophis cyanocinctus* in this protected area are the mudskippers (Gobiidae, Oxudercinae). The venom proteomes were also investigated for the same population of *Hydrophis cyanocinctus* in the Hara Biosphere Reserve of the Persian Gulf (Calvete et al. 2012; Khorjestan et al. 2016).

##### Hydrophis lapemoides

Taxon classificationAnimaliaSquamataElapidae

(Gray, 1849)

Common names: English – Persian Gulf Sea Snake; Farsi – Mâr-e daryâï-ye khalij-e fârs

Figures 4d , 21 , 22

Aturia lapemoides Gray, 1849: 46.
Distira lapemidoides -Werner 1895: 20. -Boulenger 1896: 297.
Hydrophis lapemoides -Smith 1926: 86. -Kennedy 1937: 748. -Volsøe 1939: 19. -Corkill and Cochrane 1965: 494. -Joger 1984: 33. -Leviton and Aldrich 1984: XXIV. -Gasperetti 1988: 312. -Leviton et al. 1992: 123. -Rasmussen 1993: 97. -Carpenter et al. 1997: 247. -Firouz 1999: 192. -Latifi 2000: 340. -Baldwin and Gardner 2005: 249. -Firouz 2005: 209. -Soorae et al. 2006: 109. -Egan 2007: 148. -Rastegar-Pouyani et al. 2008: 20. -Safaei and Esmaili 2009: 45. -Soorae et al. 2010: 535. -Safaei-Mahroo et al. 2015: 282.
Chitulia lapemoides -Kamali 2013: 236.

###### Material examined.

**Gulf of Oman**: 1 specimen, Jask and Ras-Meydani, depth 18–50m [(ZMSBUK.HD.40), TL 775, SVL 702, HL 19, HW 9.2, GL 11.7, SNL 3.4, NEL 2.4, ND 30, GBD 65, NSL 7, NSR 30, BSR 43, NB 45], October and November 2013, collector: M. Rezaie-Atagholipour.

###### Diagnosis.

Head slightly small (Figure 22); [8 supralabials, second in contact with prefrontal, third and fourth or third, fourth and fifth touch eye (Gasperetti 1988)] (Figure 21); body elongate but not markedly slender anteriorly (Figure 22); [290–404 ventrals (Volsøe 1939)], slightly distinguishable from adjacent scales (Figure 4d); body scales in thickest part of body more or less quadrangular or hexagonal in shape and juxtaposed; [29–31 scale rows on neck, 41–46 on body (Volsøe 1939)].

###### Coloration.

Body olive-whitish, dirty white, darker dorsally and paler ventrally; [41–55 (Volsøe 1939)] black rings broader dorsally, or broader bands tapering to points on the sides, on the body and tail (Figure 22a); head black in juveniles, usually with a yellow horseshoe-shaped mark above; adults with head sometimes of same color as body without the horseshoe-shaped mark.

###### Size.

TL 775 mm (n = 1); [n = 8, mean TL 781 mm, maximum TL 895 mm (Volsøe 1939)].

###### General distribution.

Indian Ocean, from the Persian Gulf to Malay Archipelago (Minton 1966; Rasmussen 1987).

###### IUCN Red List Category.

Least concern (IUCN 2016).

###### Remarks.

*Hydrophis lapemoides* may be easily misidentified with juveniles *Hydrophis cyanocinctus* in the Persian Gulf and Gulf of Oman (for more details see remarks on *Hydrophis cyanocinctus*). Rasmussen (1993) suggested that *Hydrophis lapemoides* is very abundant in the Persian Gulf. Gasperetti (1988) mentioned that *Hydrophis lapemoides* along with *Hydrophis cyanocinctus* are the most abundant sea snakes in both gulfs. We however could catch only one specimen during our field surveys, which was collected from Jask in the western Gulf of Oman.

#### *Microcephalophis* Lesson, 1834

##### Microcephalophis gracilis

Taxon classificationAnimaliaSquamataElapidae

(Shaw, 1802)

Common names: English – Graceful Small-headed Sea Snake, Slender Sea Snake; Farsi – Mâr-e daryâï-ye sarkuchak-e barâzandeh

Figures 23 , 24

Hydrus gracilis Shaw, 1802: 560.
Microcephalophis gracilis gracilis -Smith 1926: 121. -Kennedy 1937: 748. -Volsøe 1939: 25. -Corkill and Cochrane 1965: 494. -Joger 1984: 35. -Gasperetti 1988: 320.
Microcephalophis gracilis -Corkill 1932: 51. -Carpenter et al. 1997: 248. -Baldwin and Gardner 2005: 251. -Egan 2007: 160. -Safaei and Esmaili 2009: 45.
Hydrophis gracilis gracilis -Leviton and Aldrich 1984: XXIV. -Firouz 2005: 209.
Hydrophis gracilis -Leviton et al. 1992: 122. -Firouz 1999: 192. -Latifi 2000: 339. -Rastegar-Pouyani et al. 2008: 20. -Soorae et al. 2010: 535. -Kamali 2013: 239. -Safaei-Mahroo et al. 2015: 282.

###### Material examined.

**Gulf of Oman**: 11 specimens, Beris and Pasa-Bandar, depth 20–40m [(ZMSBUK.HD.31), TL 968, SVL 878, HL 13.1, HW 5.3, GL 7.7, SNL 3.1, NEL 1.1, ND 24, GBD 66, NSL 5, NSR 19, BSR 33, NV 237, NB 21; (ZMSBUK.HD.32), TL 830, SVL 757, HL 116, HW 4.4, GL 7, SNL 2.7, NEL 1.2, ND 19, GBD 66, NSL 6, NSR 18, BSR 31, NV 269, NB 43; (ZMSBUK.HD.33), TL 978, SVL 893, HL 13.1, HW 5.9, GL 7.8, SNL 3.4, NEL 1.9, ND 20, GBD 54, NSL 6, NSR 15, BSR 29, NV245, NB 59; (ZMSBUK.HD.34), TL 900, SVL 821, HL 12.3, HW 5.2, GL 7.9, SNL 2.8, NEL 1.4, ND 23, GBD 67, NSL 6, NSR 18, BSR 29, NV 253, NB 55; (ZMSBUK.HD.35), TL 893, SVL 821, HL 12.1, HW 6.3, GL 9, SNL 3, NEL 1.8, ND 20, GBD 55, NSL 6, NSR 17, BSR 31, NV 223, NB 20; (ZMSBUK.HD.36), TL 900, SVL 820, HL 12.2, HW 5.1, GL 9, SNL 3.6, NEL 1.7, ND 22, GBD 58, NSL 6, NSR 18, BSR 31, NV 231, NB 51; (ZMSBUK.HD.37), TL 957, SVL 870, HL 12.5, HW 5.7, GL 8.5, SNL 3.5, NEL 1.4, ND 20, GBD 50, NSL 6, NSR 19, BSR 32, NV 264, NB 46; (ZMSBUK.HD.38), TL860, SVL 787, HL 12.3, HW 5.4, GL 7.7, SNL 3, NEL 1.5, ND 20, GBD 60, NSL 6, NSR 19, BSR 29, NV 229, NB 27; (ZMSBUK.HD.39), TL 935, SVL 847, HL 12.8, HW 5.7, GL 8, SNL 3.4, NEL 2.2, ND 22, GBD 78, NSL 6, NSR 19, BSR 30, NV 229, NB 41; (ZMSBUK.HD.46), TL 827, SVL 752, HL 12.7, HW 5.6, GL 9, SNL 3.4, NEL 1.5, ND 18, GBD 62, NSL 6, NSR 19, BSR 30, NV 242, NB 48; (ZMSBUK.HD.54), TL 922, SVL 837, HL 13.2, HW 5.9, GL 8.9, SNL 3.2, NEL 1.4, ND 20, GBD 67, NSL 6, NSR 19, BSR 31, NV 246, NB 53], August 2013, collector: M. Rezaie-Atagholipour.

###### Diagnosis.

Head extremely small (Figure 24); 5–6 supralabials, second usually in contact with prefrontal, third and fourth touch eye (Figure 23a); neck markedly slender; body elongate, markedly slender anteriorly (Figure 24d); 15–20 scale rows on neck, 20–33 on body [18–19 and 31 (Volsøe 1939)]; 223–269 ventrals [232–269 (Volsøe 1939)].

###### Coloration.

Gray to dark gray dorsally, gray-whitish ventrally, darker anteriorly than posteriorly; 20–59 black bands broader dorsally; bands on necks and anterior part of body are black and merge each other only ventrally or ventrally and dorsally (Figure 24d); bands on the posterior part of the body are paler, or sometimes absent.

###### Size.

Mean TL 906 mm, maximum 978 mm (n = 11); [n = 9, mean TL 865.5 mm, maximum TL 1030 mm (Volsøe 1939)].

###### General distribution.

Indo- West Pacific, from the Persian Gulf to Australia (David and Ineich 1999).

###### IUCN Red List Category.

Least concern (IUCN 2016).

###### Remarks.

*Microcephalophis gracilis* and the other Small-headed Sea Snake, *Microcephalophis cantoris*, recorded here for the first time in the area (see below), are easily distinguishable from other species in the region by having an extremely small head and slender neck. However, these two species may be confused with each other at a glance. *Microcephalophis gracilis* is distinguishable from *Microcephalophis cantoris* by having less number of ventrals (223–269 versus 404–468 in *Microcephalophis cantoris*). All material examined in this study was collected from eastern Gulf of Oman. Volsøe (1939) however recorded material from the western Gulf of Oman and western Persian Gulf.

##### Microcephalophis cantoris

Taxon classificationAnimaliaSquamataElapidae

(Günther, 1864)

Common names: English – Gunther’s Sea Snake; Farsi – Mâr-e daryâï-ye gunder

Figures 25 , 26

Hydrophis cantoris Günther, 1864: 374.

###### Material examined.

**Gulf of Oman**: 1 specimen, Jask and Ras-Meydani, depth 18–50m [(ZMSBUK.HD.), TL 1124, SVL 992, HL 33.5, HW 12.3, GL 20.2, SNL 5.1, NEL 4.4, ND 51, GBD 101, NSL 6, NSR 24, BSR 41, NV 446], October and November 2013, collector: M. Rezaie-Atagholipour.

###### Diagnosis.

Head extremely small and pointed (Figure 26); third supralabial usually in contact with prefrontal (Figure 25a); body elongate, markedly slender anteriorly; neck markedly slender (Figure 26d); 24 scale rows on neck [23–25 (rarely 21) (Leviton et al. 2003)] and 41 on body; 446 ventrals [404–468 (Leviton et al. 2003)].

###### Coloration.

Head yellowish; neck and body dark olive dorsally, yellowish ventrally; dorsal portion of body uniform in color and not banded; neck with the rings paler dorsally and black ventrally (Figure 26d); [Dark greenish olive dorsally, yellowish ventrally; ventrals blackish; dorsal portion of body uniform in color and not banded; slender part of body with 20–28 blackish bands merging dorsally and ventrally (Günther 1864)].

###### Size.

TL 1124 mm (n = 1) [1450 mm in males and 1880 mm in females (Leviton et al. 2003)]

###### General distribution.

Indian Ocean, from the Gulf of Oman to Malay Archipelago.

###### IUCN Red List Category.

Data deficient (IUCN 2016).

###### Remarks.

It is the first record of *Microcephalophis cantoris* in this area. By this record, westernmost extent of *Microcephalophis cantoris* expands from Pakistan to the Gulf of Oman. Safaei and Esmaili (2009) recorded four specimens of this species from the same area localities (Jask), but they have presented neither morphological data nor descriptions of the specimens. Furthermore, their specimens were not deposited in any public museum or collection and we couldn’t find them for further morphological examination. Regarding the single specimen examined herein, the shape of the fangs was found to be unique: elongated fangs markedly protruding outside the lower jaw when mouth is closed. This unusual characteristic could be age-dependent or likely a specific character which was overlooked by other authors. Further studies on this specimen are much recommended.

## Supplementary Material

XML Treatment for Hydrophis schistosus

XML Treatment for Hydrophis viperinus

XML Treatment for Hydrophis curtus

XML Treatment for Hydrophis platurus

XML Treatment for Hydrophis spiralis

XML Treatment for Hydrophis ornatus

XML Treatment for Hydrophis cyanocinctus

XML Treatment for Hydrophis lapemoides

XML Treatment for Microcephalophis gracilis

XML Treatment for Microcephalophis cantoris
